# Role of a chalcone isomerase-like protein in flavonoid biosynthesis in *Arabidopsis thaliana*

**DOI:** 10.1093/jxb/erv413

**Published:** 2015-09-07

**Authors:** Wenbo Jiang, Qinggang Yin, Ranran Wu, Guangshun Zheng, Jinyue Liu, Richard A. Dixon, Yongzhen Pang

**Affiliations:** ^1^Key Laboratory of Plant Resources and Beijing Botanical Garden, Institute of Botany, Chinese Academy of Sciences, Beijing 100093, China; ^2^University of Chinese Academy of Sciences, Beijing 100049, China; ^3^Department of Biological Sciences, University of North Texas, 1155 Union Circle, 305220 Denton, TX, USA

**Keywords:** *Arabidopsis*, chalcone isomerase-like, flavonoids, flavonols, proanthocyanidins, seed coat.

## Abstract

Flavonoids are important natural products for plant defence and human health. Although almost all the flavonoid pathway genes have been well-documented by biochemical and/or genetic approaches, the role of the *Arabidopsis chalcone isomerase-like* (*CHIL*) gene remains unclear. Two *chil* mutants with a seed colour similar to that of wild-type *Arabidopsis* have been identified here, but in sharp contrast to the characteristic *transparent testa* seed phenotype associated with other known flavonoid pathway genes. *CHIL* loss-of-function mutations led to a strong reduction in the proanthocyanidin and flavonol levels in seeds, but not in the anthocyanin levels in leaves. *CHIL* over-expression could partially recover the mutant phenotype of the *chil* mutant and increased both proanthocyanidin and flavonol accumulation in wild-type *Arabidopsis*. However, the *CHIL* gene could not rescue the mutant phenotype of *TT5* that encodes the intrinsic chalcone isomerase in *Arabidopsis*. Parallel phenotypical and metabolic analyses of the *chil*, *tt5, chs*, and *f3h* mutants revealed that, genetically, CHIL functions at the same step as TT5. Moreover, it is demonstrated that CHIL co-expresses, co-localizes, and interacts with TT5 in *Arabidopsis* for flavonoid production. Based on these genetic and metabolic studies, it is concluded that CHIL functions with TT5 to promote flavonoid production, which is a unique enhancer in the flavonoid pathway.

## Highlight

CHIL is essential for the accumulation of proanthocyanidins and flavonols in seeds of *Arabidopsis* and it functions as a unique enhancer in the flavonoid pathway.

## Introduction

Flavonoids are a large group of natural products that are widely present in the leaves, flowers, fruits, and seeds of various plants. Based on their basic chemical structures, flavonoids can be further divided into different subgroups, including the three major subgroups of flavonols, anthocyanins, and proanthocyanidins (PAs) (Winkel-Shirley, 2001; Lepiniec *et al.*, 2006). Flavonoids play important roles in many aspects of plant development and response to stress, including UV light protection, auxin transport, and resistance to pathogens (Lattanzio *et al.*, 2006; Ferreyra *et al.*, 2012; Hassan and Mathesius, 2012). Meanwhile, flavonoids are also known to have beneficial effects on human health, such as anti-viral, antioxidant, and anticancer activities and they are also known to lower the incidence of cardiovascular disease, obesity, and diabetes (Yao *et al.*, 2004; Lee *et al.*, 2007; Kozlowska and Szostak-Wegierek, 2014). Given their importance in both plant physiology and human nutrition, it is not surprising that there has been a great deal of research efforts focused on the flavonoid biosynthetic pathway in a range of plant species over the last several decades.

**Fig. 1.**
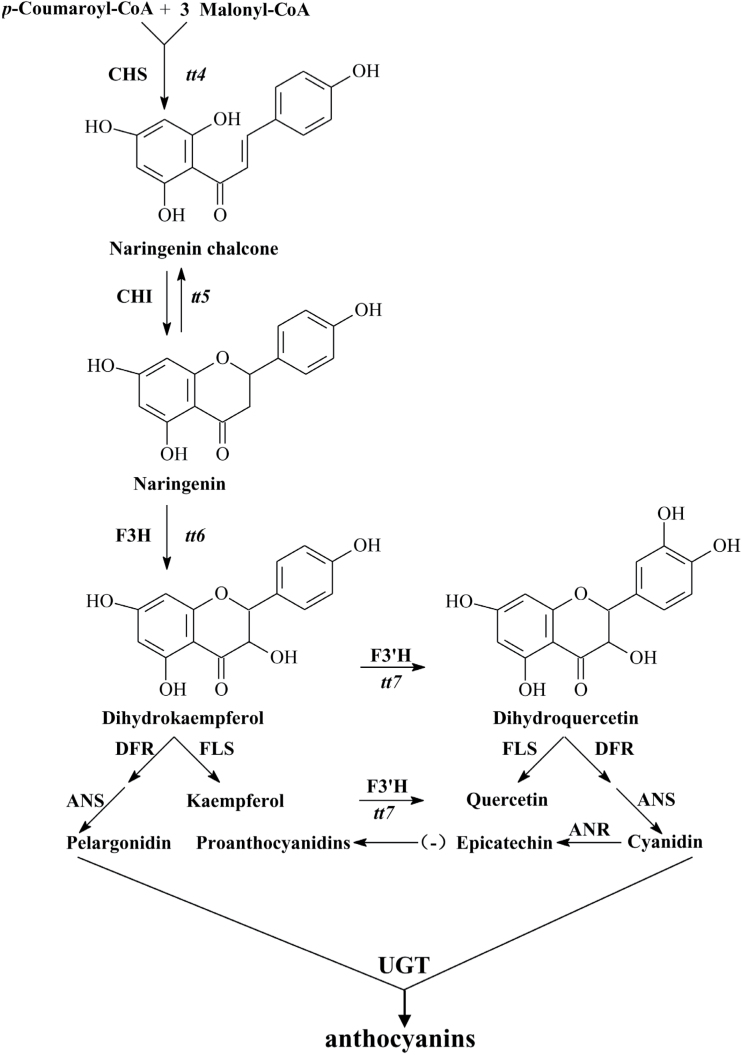
Flavonoid biosynthetic pathway in *A. thaliana*. Abbreviations: ANR, anthocyanidin reductase; ANS, anthocyanidin synthase; CHI, chalcone isomerase; CHS, chalcone synthase; DFR, dihydroflavonol 4-reductase; F3H, flavanone 3-hydroxylase; F3′H, flavanone 3′-hydroxylase; FLS, flavonol synthase; *tt*, the corresponding *transparent testa* mutants; UGT, UDP-glucosyltransferase.

In the core flavonoid pathway, chalcone synthase (CHS, encoded by the *TT4* locus) catalyses the first committed step to form 4,2′,4′,6′-tetrahydroxychalcone (naringenin chalcone), by the stepwise condensation of three molecules of malonyl-CoA with one molecule of *p*-coumaroyl-CoA. Chalcone isomerase then catalyses the isomerization of naringenin chalcone into 5,7,4′-trihydroxyflavanone (naringenin), which is considered to be the earliest intermediate with the flavonoid core. Naringenin can be hydroxylated by flavanone 3-hydroxylase (F3H) to form dihydrokaempferol, and dihydrokaempferol can be further hydroxylated by flavanone 3′-hydroxylase (F3′H) to form dihydroquercetin. These intermediates are further converted to flavonols, anthocyanins, and PAs through distinct branches of the flavonoid pathway (Fig. 1). Flavonols, anthocyanins, and PAs are three major subgroups of flavonoid molecules known to occur in *Arabidopsis thaliana*, where they accumulate in an organ-specific manner (Lepiniec *et al.*, 2006). Among them, flavonols are accumulated in almost all organs, including leaves and seeds, whereas anthocyanins are present in all organs with the exception of the immature seeds. Unlike flavonols and anthocyanins, PAs and their precursor (-)-epicatechin are specifically accumulated in the seed coat (Lepiniec *et al.*, 2006; Routaboul *et al.*, 2006; Saito *et al.*, 2013). PAs can react with *p*-dimethylaminocinnamaldehyde (DMACA) to form a blue to purple stain (Li *et al.*, 1996). Typically, mutations in the genes encoding the enzymes of the core flavonoid pathway usually lead to *transparent testa* seed phenotypes in *Arabidopsis* due to the lack of oxidized flavonoid compounds (mainly PAs).

The chalcone isomerase-like protein (CHIL, At5g05270) is probably the least well-documented among the potential functional proteins in the flavonoid pathway of *Arabidopsis*; only a few characteristics of CHIL have been reported, often in parallel with studies of TT5 (At3g55120). Both CHIL and TT5 belong to the CHI super-family that comprises four subclasses in *Arabidopsis*. Type I CHI proteins are ubiquitous in vascular plants and are responsible for the formation of 5,7,4′-trihydroxyflavanone. Type II CHIs are mostly found in leguminous plants and catalyse the formation of 5-deoxy(iso)flavonoids (Shimada *et al.*, 2003; Ralston *et al.*, 2005; He *et al.*, 2011). Both Type I and II CHI proteins are bona fide enzymes with catalytic activity. Type III CHIs, of which there are three in *Arabidopsis*, were recently shown to be fatty acid-binding proteins (FAPs) that are present widely in land plants and green algae (Ngaki *et al.*, 2012). There is only one member of Type IV CHI proteins in *Arabidopsis* known as CHIL, and it shares less than 30% identity with TT5 at the amino acid level. Type IV CHI proteins are only found in land plants (Ralston *et al.*, 2005; Ngaki *et al.*, 2012).

In a previous study that used comprehensive flavonoid profiling and transcriptome co-expression analyses, the *CHIL* gene was found to be co-expressed with 9 out of the 19 fully characterized flavonoid pathway genes (Yonekura-Sakakibara *et al.*, 2008), including *Anthocyanidin Reductase* (*ANR*), a PA-specific pathway gene (Xie *et al.*, 2003). Similarly, in several other studies, *CHIL* was also found to be co-expressed with one or more flavonoid pathway genes, including *Flavonol Synthase* (*FLS*) and/or *TT5* (Ma, 2002; Oravecz *et al.*, 2006; Pan *et al.*, 2009). These data indicated that *CHIL* is an expressed gene in *Arabidopsis* and that it is potentially associated with flavonoid biosynthesis. To date, many of the known mutants of flavonoid pathway genes, with the exception of *FLSs* (Wisman *et al.*, 1998) and glycosyltransferase genes required for the formation of flavonol glycosides and anthocyanins, exhibited a *transparent testa* seed phenotypes with a yellow or pale brown seed coat colour (Saito *et al.*, 2013). However, no *transparent testa* mutation has been shown to be associated with a loss-of-function of *CHIL* and, importantly, no *in vitro* catalytic activity has been reported for CHIL (Jez *et al.*, 2000; Ngaki *et al.*, 2012). Therefore, at present, neither the genetic nor the biochemical role(s) of CHIL is clear.

In the present study, it was found that the expression level of the *CHIL* gene was down-regulated in a *tt2* mutant that failed to produce PAs, implying that *CHIL* is involved in PA biosynthesis. Mutants for *CHIL* in *Arabidopsis* were further identified using a reverse genetics approach; interestingly, the seeds exhibited a brown seed colour phenotype similar to the wild-type. *CHIL* was not able to rescue the *tt5* phenotype, but it did enhance the accumulation of flavonol and PA compounds in the presence of *TT5*. Moreover, the CHIL protein co-expresses and co-localizes with TT5, which implies that CHIL may enhance flavonoid production by co-operation with TT5 in *Arabidopsis*. Our results indicate a functional role for *CHIL* gene with a distinct characteristic different from other known genes in the flavonoid pathway.

## Materials and methods

### Plant materials and growth conditions

The *Arabidopsis* mutants *chil-1* (SALK_096551C, Alonso *et al.*, 2003), *chil-2* (CS418063, Rosso *et al.*, 2003), *chi* (*tt5*, SALK_034145, Alonso *et al.*, 2003), *chs* (*tt4*, CS66119, Zhang *et al.*, 2010), and *tt2* (SALK_005260 and CS83, Koornneef, 1981; Alonso *et al.*, 2003) were obtained from the Arabidopsis Biological Resource Center (The Ohio State University, Columbus, OH, USA). The *tt8* mutant (SALK_063334, Alonso *et al.*, 2003) was a gift from Professor Lixi Jiang (Zhejiang University, Hangzhou, China). The *ttg1-9* (Pang *et al.*, 2009) and *tt2* (CS83) mutants are in the Landsberg erecta background, other mutants and transgenic *Arabidopsis* plants are in the Col-0 background. *Arabidopsis* seeds were surface-sterilized with 10% bleach and stratified on plates with 1/2 strength Murashige and Skoog (1/2 MS) medium containing 5% (w/v) sucrose. For the observation of anthocyanins in 4-d-old seedlings, the seeds were kept in the dark at 4 °C for 3 d and then transferred to a tissue culture room at 22 °C under constant light (150 μmol photons m^–2^ s^–1^) for 4 d. For the flavonoid analysis and DMACA staining, plants were grown at 22 °C with 16/8h light/dark cycles. The seeds were harvested when the plants were completely mature and were dried at 37 °C for 7 d after harvest. For the analysis of anthocyanin content, the seeds were stratified on plates with 1/2 MS medium containing 1% (w/v) sucrose in the dark at 4 °C for 3 d, and then transferred to a tissue culture room at 22 °C under constant light (40 μmol photons m^–2^ s^–1^) for 14 d. These seedlings were transferred to plates with 1/2 MS medium containing 12% (w/v) sucrose and grown under constant light (80 μmol photons m^–2^ s^–1^) for 3 d (Yonekura-Sakakibara *et al.*, 2012). For Norflurazon treatment, seeds were stratified on plates with 1/2 MS medium containing 100 μM Norflurazon in the dark at 4 °C for 3 d, and grown at 22 °C with 16/8h light/dark cycles for 4 d. For the detection of flavonol content, sterile seeds were grown on MS medium lacking sucrose. The plates were kept for 5 d in the dark at 4 °C and the seedlings were grown in a tissue-culture room at 22 °C under constant light (100 μmol photons m^–2^ s^–1^) for 21 d.

### Microarray hybridization and data analysis

The seedlings of wild-type (L*er*) and *tt2* (CS83) *Arabidopsis* were grown under the conditions described above. The flowers were tagged with different coloured tape to indicate the days after pollination. The siliques (6 d after pollination) were conducted for L*er* and CS83 in the microarray experiment with three independent biological replicates. Probe labelling and chip hybridization followed the procedures suggested by the manufacturer (http://www.affymetrix.com/support/technical/manual/expr-ession_manual.affx). Statistical analysis of microarray data and gene selection followed the procedures detailed in Pang *et al.* (2008). All microarray data have been deposited in ArrayExpress (http://www.ebi.ac.uk/microarray-as/ae/) under the accession number E-MTAB-3659.

### Identification of the *chil* T-DNA insertion mutants

Identification of the *Arabidopsis* T-DNA insertion mutants was performed as previously described (Jiang and Yu, 2009). Homozygous plants of the *chil* mutant were identified by two round PCRs. In the first round PCR, a pair of *CHIL*-specific primers U1 (forward, 5′-TGAGTGGTTGTGGACTGAGTG-3′) and U2 (reverse, 5′-AAGTGATGATCTGTGGAGGA-3′) were designed to anneal to a location outside the T-DNA insertion in order to ensure the production of a band with an expected size in the non-homozygosity. In the subsequent PCR, the T-DNA border primer (5′-ATTTTGCCGATTTCGGAAC-3′) and primer U1 were used. Homozygous *chil-2* T-DNA insertion lines were obtained by PCR screening using primer U2 (forward, 5′-AGTTCACTGCGATCGGAGTT-3′) and L2 (reverse, 5′-TCCTCAGCCAAACGATCTCT-3′). To confirm the nature and location of the T-DNA insertion, the PCR products were sequenced. To remove additional T-DNA loci or mutations from the *chil* mutants, backcrosses to wild-type plants were performed, and plants homozygous for the T-DNA insertion were identified again.

### Gene expression analysis

**Fig. 2.**
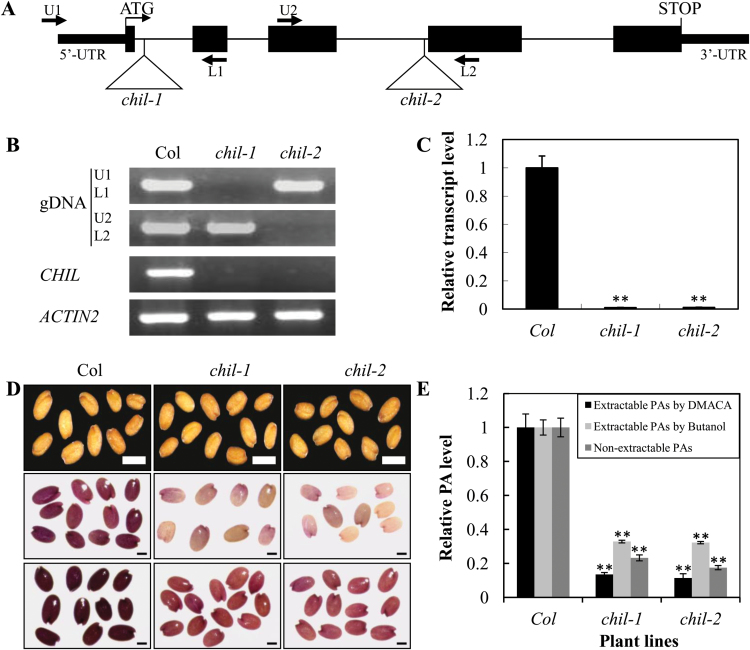
Characterization of the two *chil* mutants. (A) Diagram of *CHIL* gene structure [thick lines, untranslated regions (UTRs); thin lines, introns; black boxes, exons] with the relative position of the two T-DNA insertions corresponding to the *chil-1* and *chil-2* mutants. The positions of the U1/L1 and U2/L2 primer pairs used in the screening of homozygous lines are shown, respectively. (B) Molecular characterization of the two *chil* mutant lines at the genomic (confirmation by using U1/L1 and U2/L2 primer pairs for the *chil-1* and *chil-2* lines, respectively) and transcript levels (by using semi-quantitative RT-PCR). (C) Relative transcript levels of the *CHIL* gene in 4-d-old siliques detected by qRT-PCR analysis. Data are presented as mean ±SD, Student’s *t* test (*n*=3, *P <0.05, **P <0.01). (D) Seed phenotype of the *chil* mutants, compared with the wild-type *Arabidopsis* ecotype Col. Seed phenotype before staining with DMACA (upper). Bars=0.5mm. Seed phenotype after staining with DMACA for 2 d (middle) and 20 d (lower), respectively. Bars=0.2mm. (E) Relative proanthocyanidin levels in seeds of Col and *chil* mutants detected by different methods. The extractable PAs measured by DMACA (2.64mg epicatechin equivalence g^–1^) and by butanol (1.08mg B2 equivalence g^–1^); non-extractable PAs (2.50mg B2 equivalence g^–1^) in Col were set as a value of 1.0, respectively. Data are presented as mean ±SD, Student’s *t* test (*n*=3, *P <0.05, **P <0.01). (This figure is available in colour at *JXB* online.)

Total RNA preparation, first-strand cDNA synthesis, and qRT-PCR were performed as previously described (Jiang *et al.*, 2013), with minor modifications. The DNase I-treated total RNA (2 μg) was denatured and subjected to reverse transcription using Moloney Murine Leukemia Virus Reverse Transcriptase (Promega). Semi-quantitative RT-PCR was carried out on Applied Biosystems Veriti Thermal Cycler. The PCR conditions were 94 °C for 3min; 28 cycles of 94 °C for 20 s, 60 °C for 20 s, and 72 °C for 20 s; followed by a final extension of 72 °C for 10min. Quantitative RT-PCR analyses were carried out on Mx3000P (Stratagene) by using the SYBR Green reagent (Kapa) according to the manufacturer’s instructions. The *ACTIN2* gene was used as a control in the qRT-PCR in Fig. 2. The *UBQ10* gene was used as a control in the other qRT-PCRs. The primer sequences used for qRT-PCR were all listed in Supplementary Table S2 at *JXB* online. Data were calculated from three biological replicates, and each biological replicate was examined in triplicate.

### Generation of transgenic plants

To produce constitutively *CHIL*-expressing *Arabidopsis* plants, the 630-bp CDS (coding sequence) fragment was amplified by PCR with primers CHILCF and CHILR and then cloned into the Gateway Entry vector pENTR/D/TOPO (Invitrogen) and confirmed by sequencing. For stable transformation by *Agrobacterium tumefaciens*, the CDS of *CHIL* was first transferred into the Gateway plant transformation destination vector pB2GW7 (Karimi *et al.*, 2002) by performing an LR recombination reaction with the resulting entry vector pENTR-CHIL according to the manufacturer’s instructions (Invitrogen). The resulting pB2GW7-CHIL vector was then transformed into *A. tumefaciens* strain GV3101 for *Arabidopsis* transformation. In the same way, the 1742-bp promoter fragment of *CHIL* was cloned into entry vector with primers CHILPF and CHILPR and transferred to vector pBGWFS7 for GUS staining. The resulting vector pBGWFS7-CHIL was transformed into *A. tumefaciens* strain GV3101 for *Arabidopsis* transformation. Transformation of *Arabidopsis* was performed by the floral dip method (Clough and Bent, 1998). T2 generation seeds were germinated on plates with MS containing 2.5mg ml^–1^ phosphothricin, and the resistant seedlings were transferred to soil to obtain homozygous T3 generation seeds. For more detailed phenotypic analysis, three independent T3 generation homozygous lines were used for further analysis.

### Purification and detection of recombinant proteins

The recombinant CHIL and TT5 proteins were obtained by cloning its ORF into the expression vector pQE-30 (Qiagen), followed by transformation into *E. coli* strain M15. The primers used were CHILBF and CHILSR, and TT5BF and TT5BR (see Supplementary Table S2 at *JXB* online). The soluble histidine (His)-tagged fusion protein was purified using Ni-NTA affinity resin (Qiagen) according to the manufacturer’s instruction. The reaction were conducted at 25 °C for 10min in a 100 µl-volume reaction with the following components: 50mM TRIS-HCl buffer (pH 7.6) containing 1% ethanol, 100 µM naringenin chalcone, and 10 µg recombinant protein. The reaction with the same above-mentioned components but 10 µg boiled recombinant protein was used as the control.

Enzymatic reactions were stopped with 100 µl methanol, followed by centrifugation at 12 000rpm for 30min. One-hundred microlitre samples were then run on an HPLC 1260 (Agilent) system with an Eclipse XDB-C18 reverse phase column (4.6×150mm, particle size 5 µm). Compounds were separated with a linear eluting gradient (5–70% solvent B over 30min) with solvent A (0.1% formic acid in water) and solvent B (0.1% formic acid in acetonitrile) at flow rate of 1ml min^–1^. A photodiode array detector (Agilent) was used for the detection of UV-visible absorption from 190–600nm.

### Extraction and quantification of anthocyanins, flavonols, and proanthocyanidins

**Fig. 3.**
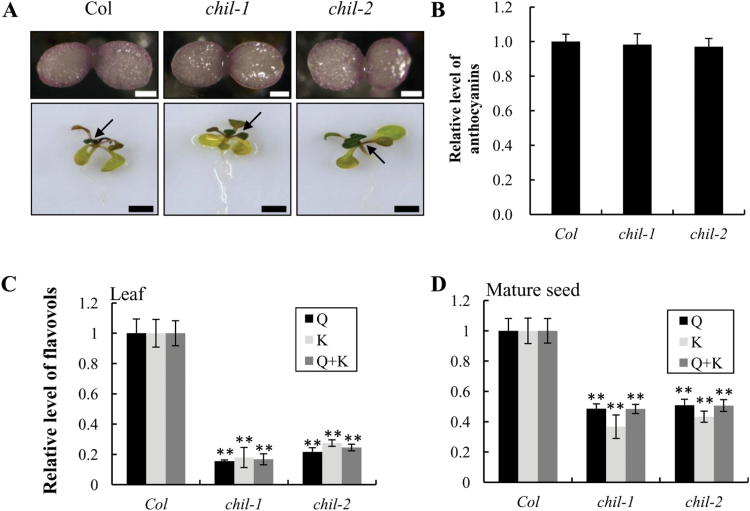
Flavonoid accumulation in the *chil* mutants. (A) Anthocyanin accumulation in the 4-d-old *Arabidopsis* seedlings (upper, bars=0.5mm) and 17-d-old seedlings (bottom, bars=5mm). (B) Relative level of anthocyanins in the 17-d-old seedlings of Col and the *chil-1* and *chil-2* mutants. The level of anthocyanins in the wild-type control Col (1.57mg g^–1^ dry weight) was set as a value of 1.0. (C, D) Relative level of the major flavonols [quercetin (Q) and kaempferol (K) aglycones] in 21-d-old seedlings (C) and mature seeds (D) of Col and the *chil-1* and *chil-2*. Levels of quercetin (4.79 μg g^–1^ dry weight and 584.27 μg g^–1^ dry weight in seedlings and seeds, respectively) and kaempferol (4.90 μg g^–1^ dry weight and 11.38 μg g^–1^ dry weight in seedlings and seeds, respectively) from wild-type control Col were set as a value of 1.0. Data are presented as mean ±SD, Student’s *t* test (*n*=3, *P <0.05, **P <0.01). (This figure is available in colour at *JXB* online.)

Anthocyanins were extracted from leaf tissue with methanol containing 0.1% HCl, followed by the addition of the same amount of water and chloroform to remove chlorophyll, and then measured spectrophotometrically at 530nm. The anthocyanin level was calculated and compared with the wild-type control Col in biological triplicates (1.57mg g^–1^ dry weight in the wild-type control Col was set as a value of 1.0 in Fig. 3B and in Supplementary Fig. S4 at *JXB* online).

**Fig. 4.**
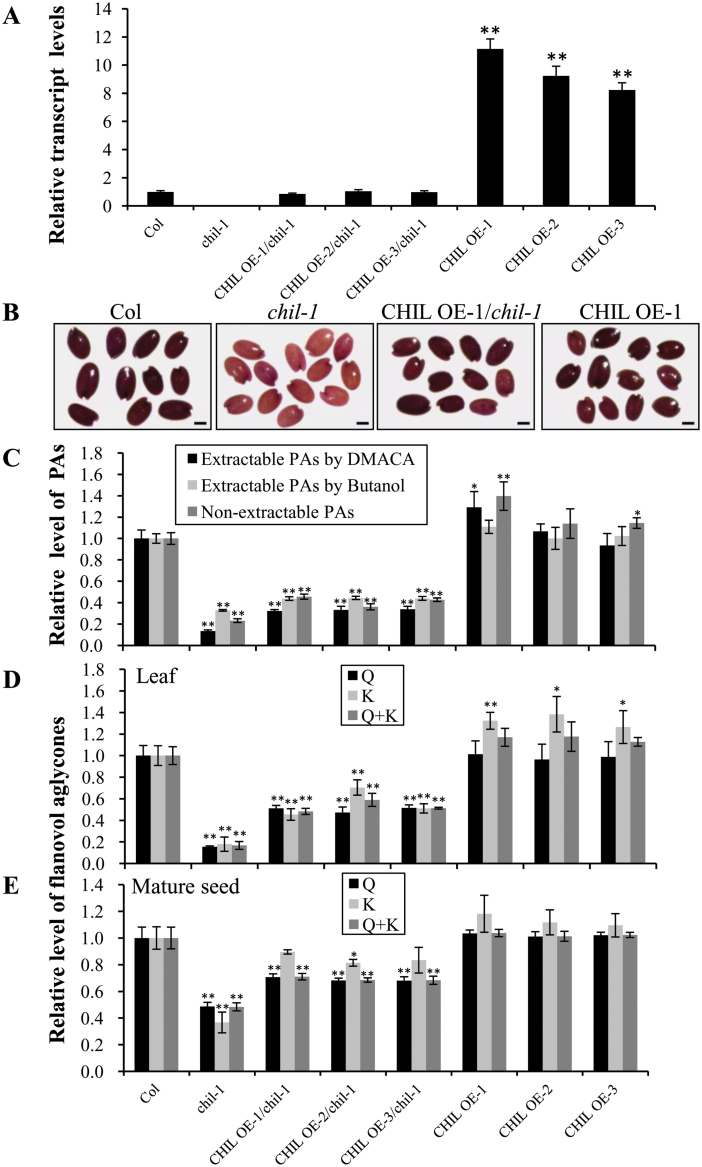
Over-expression of the *CHIL* gene in the *chil* and Col background. (A) Relative transcript levels of the *CHIL* gene in the Col wild-type, *chil-1*, three CHIL OE/*chil-1* lines, and three CHIL OE lines detected by qRT-PCR analysis. (B) Seed phenotypes of Col, *chil-1*, CHIL OE-1/*chil-1*, and CHIL OE-1 after staining with DMACA for 20 d. Bars=0.2mm. (C) Relative level of proanthocyanidins in mature seeds of Col, *chil-1*, three CHIL OE/*chil-1* lines, and three CHIL OE lines. The extractable PAs measured by DMACA (2.64mg epicatechin equivalence g^–1^) and by butanol (1.08mg B2 equivalence g^–1^); non-extractable PAs (2.50mg B2 equivalence g^–1^) in Col were set as a value of 1.0, respectively. (D, E) Relative levels of flavonols [quercetin (Q) and kaempferol (K) aglycones] in 21-d-old seedlings (D) and mature seeds (E) of Col, *chil-1*, three CHIL OE/*chil-1* lines, and three CHIL OE lines. Levels of quercetin (4.79 µg g^–1^ dry weight and 584.27 µg g^–1^ dry weight in seedlings and seeds, respectively) and kaempferol (4.90 µg g dry weight and 11.38 µg g^–1^ dry weight in seedlings and seeds, respectively) from wild-type control Col were set as a value of 1.0. (A, C, D, E) Data are presented as mean ±SD, Student’s *t* test (*n*=3, *P <0.05, **P <0.01). (This figure is available in colour at *JXB* online.)

Flavonols were extracted with 80% methanol overnight, the extract was hydrolysed with the same amount of 6 N HCl at 70 °C for 40min, followed by the addition of the same amount of methanol. Twenty to forty microlitres of the extract was run on the HPLC with the same conditions as mentioned above. Flavonols standards were purchased from the Shanghai Tongtian Biotechnology Company. Levels of quercetin (4.79 µg g^–1^ dry weight and 584.27 µg g^–1^ dry weight in seedlings and seeds, respectively) and kaempferol (4.90 µg g^–1^ dry weight and 11.38 µg g^–1^ dry weight in seedlings and seeds, respectively) from the wild-type control Col were set as a value of 1.0 in Figs 3C, D, 4D, E, and 6D, E).

For proanthocyanidin staining, mutants and wild-type seeds were stained with 0.1% DMACA reagent in methanol:6 N HCl (1:1 v/v) for different time points. Extractable proanthocyanidins were extracted from the powder of 100mg of dry seeds three times with 70% acetone containing 0.5% acetic acid, three times with chloroform, and twice with hexanes; the aqueous phase was then freeze-dried and resuspended in 70% acetone containing 0.5% acetic acid. The proanthocyanidin levels were determined by reaction with 0.2% DMACA (methanol:6 N HCl=1:1) at 640nm. The extractable PA level in seeds of the wild-type control Col (2.64mg epicatechin equivalence g^–1^) was set as a value of 1.0. The extractable PA levels were also measured by heating with butanol/HCl (95:5 v/v) at 550nm (Pang *et al.*, 2007), and the PA level (1.08mg B2 equivalence g^–1^) in the wild-type control Col was set as a value of 1.0. Non-extractable PAs were measured by heating with butanol/HCl and determining the absorption at 550nm. The non-extractable PA level in the wild-type control Col (2.50mg B2 equivalence g^–1^) was set as a value of 1.0. All the assays were conducted in triplicate, and the relative levels were compared with that of the Col control as a value of 1.0 in Figs 2E, 4C, and 6C).

### Promoter analysis with GUS staining

*Arabidopsis* tissue for GUS staining was immersed in 90% ice-cold acetone for 20min and then incubated in GUS staining solution (100mM sodium phosphate, pH 7.0; 10mM EDTA; 0.5mM potassium ferricyanide; 0.5mM potassium ferrocyanide; 1mM 5-bromo-4-chloro-3-indolyl β-d-glucuronic acid; 0.1% (v/v) Triton X-100) at 37 °C. Tissues were cleared in 70% ethanol before observation.

### Transient expression of CHIL-GFP and TT5-GFP fusion proteins in *Arabidopsis* leaf mesophyll protoplasts

The coding sequences of *CHIL* and *TT5* genes were amplified with primer pairs CHIL-1302-F/CHIL-1302-R for *CHIL* and TT5-1302-F/TT5-1302-R for *TT5*, respectively. The restriction sites *Nco*I and *Spe*I were introduced into the PCR products of both *CHIL* and *TT5*. The PCR products were digested with *Nco*I and *Spe*I, and cloned into the pCAMBIA1302 plasmid digested with the same enzymes. After confirmation by sequencing, the recombinant constructs were used in protoplast transformation, and pCAMBIA1302 was used as the positive control.

Isolation and PEG-mediated transformation of *Arabidopsis* protoplasts by the constructs mentioned above were as described by Sheen (2002). After incubation for 16-24h, GFP fluorescence in *Arabidopsis* protoplast cells was detected by laser scanning confocal microscopy using Leica TCS SP5. The emission from 500nm to 560nm was collected for GFP and from 605nm to 700nm for chlorophyll.

### *Arabidopsis* gene IDs studied

Gene IDs in the *Arabidopsis* Genome Initiative database are as follows: *ACTIN2* (At3G18780), *UBQ10* (At4G05320), *CHIL* (At5G05270), *CHS* (At5G13930), *TT5* (At3G55120), *F3H* (At3G51240), *F3′H* (At5G07990), *DFR* (At5G42800), *ANS* (At4G22880), *ANR* (At1G61720), *FLS* (At5G08640), *TT2* (At5G35550), *TT8* (At4G09820), and *TTG1* (At5G24520), *UGT79B1* (At5G54060), *UGT84A2* (At3G21560), and *UGT78D2* (At5G17050).

## Results

### Identification and characterization of the *chil* mutants in *Arabidopsis*

TT2, an R2-R3 MYB type transcription factor, is a master regulator of PA biosynthesis in the seed coat of *Arabidopsis* (Nesi *et al.*, 2001). In order to screen for genes involved in PA biosynthesis, a *tt2* mutant line (CS83) with a pale yellow seed colour phenotype was used for global gene expression analysis using the *Arabidopsis* Affymetrix microarray. The result revealed that 255 probe sets were down-regulated by 2.0-97.4-fold in the *tt2* mutant (see Supplementary Table S1 at *JXB* online). As expected, several PA-specific pathway genes, including *ANR* (At1g61720) and *AHA10* (At1g17260) were down-regulated (see Supplementary Table S1 at *JXB* online). Interestingly, *CHIL* expression was reduced by 2.3-fold in the mutant, indicating that CHIL may be involved in PA biosynthesis.

To investigate whether the uncharacterized *CHIL* gene was indeed involved in PA biosynthesis, two independent T-DNA insertion mutant alleles, *chil-1* and *chil-2,* were obtained from ABRC. It was further confirmed by PCR screening that the T-DNAs were inserted into the first and the third introns of the *CHIL* gene in the *chil-1* and the *chil-2* mutants, respectively (Fig. 2A), disrupting transcription of the *CHIL* gene. This was also confirmed with PCR using gene-specific primer pairs (Fig. 2B). No *CHIL* transcripts were detected by reverse transcription PCR or quantitative Real Time PCR (qRT-PCR) in homozygous genotypes of either mutant line (Fig. 2B, C).

The seed colour of the two mutant lines was essentially the same as that of the wild-type Col (Fig. 2D, upper panel), a finding in sharp contrast to the pale yellow seed colour phenotypes of the mutants of many other flavonoid pathway genes. The fact that the seed coats were the same colour suggests insignificant changes in levels of the major seed coat pigments. However, when stained with DMACA, a dye that is specific for PAs, seeds of the *chil-1* and *chil-2* mutants exhibited a lighter blue-purple colour than the wild-type seed (Fig. 2D, middle and lower panels). This strongly suggests that the *chil* mutants accumulate lower levels of PA compounds than does the wild-type; this result was further confirmed by a determination of the extractable PA content by either the DMACA or acid butanol hydrolysis methods (Fig. 2E). In addition, the non-extractable PA levels were greatly reduced in the mutant, to about 20% of the level of the wild-type control (Fig. 2E).

To evaluate whether flavonoid compounds other than PAs are affected by a loss-of-function of *CHIL*, seedlings of the *chil-1* and *chil-2* mutants were observed for anthocyanin accumulation compared with the wild-type control. Anthocyanins normally accumulated in the hypocotyls of 4-d-old seedlings of both the wild-type and the mutants under normal growth conditions (Fig. 3A, upper panels). The accumulation of anthocyanins is known to be stress inducible. To visualize the accumulation of anthocyanins in *Arabidopsis* better, 17-d-old seedlings were therefore grown under stress conditions (increased sucrose concentration in the growth medium and higher light intensity) as previously described by Yonekura-Sakakibara *et al.* (2012). These experiments revealed that the two *chil* mutants accumulated similar levels of anthocyanins in the true leaves as did the wild-type (Fig. 3A, lower panels). Spectrometric quantification further confirmed that the anthocyanin levels in the two *chil* mutants did not differ significantly from the level of the wild-type (Fig. 3B). These results indicated that CHIL does not affect the accumulation level of anthocyanins in *Arabidopsis* leaves.

Flavonols are among the major flavonoid compounds accumulating in leaves and seeds of *Arabidopsis*. To evaluate whether *CHIL* loss-of-function mutants affected flavonol accumulation, flavonoids extracted from leaves and seeds of mutant and wild-type plants were hydrolysed and analysed by HPLC. Levels of the two major flavonol aglycones, quercetin and kaempferol, were decreased greatly (down to 17-25%) in the *chil* mutants in both leaves and mature seeds compared with the wild-type (Fig. 3C, D; see Supplementary Fig. S1A–C at *JXB* online), indicating that CHIL affects flavonol accumulation in both seeds and leaves of *Arabidopsis*.

### *CHIL* is a functional gene that enhances flavonoid production in *Arabidopsis*

To verify whether *CHIL* is functional *in vivo*, and to confirm that the phenotype of the *chil-1* mutant was indeed the consequence of loss-of-function of the *CHIL* gene, *CHIL* was over-expressed in the *chil-1* mutant background. PCR-positive transgenic lines were further evaluated by qRT-PCR to determine the transcript level of the *CHIL* gene in the transgenic lines. The transcript levels of *CHIL* in the three lines over-expressing the *CHIL* gene (in the *chil-1* background) were all increased to a level that is comparable with the wild-type control (Fig. 4A). The *CHIL* over-expressing lines in the *chil-1* background produced brown coloured seeds, which was the same as the wild-type (see Supplementary Fig. S2A at *JXB* online). Further analysis showed that the seed appeared to accumulate a similar level of PAs as visualized by DMACA staining (Fig. 4B; see Supplementary Fig. S2B at *JXB* online). The extractable and non-extractable PA levels in the mature seeds of the over-expression lines were all increased (Fig. 4C), but were not restored to the level found in the wild-type, which may be because the promoter used (the cauliflower mosaic virus 35S promoter) for the construct may be less strong than the endogenous promoter. Similarly, the levels of flavonol aglycones in the *CHIL* over-expression lines were increased, but not to levels comparable with those of the wild-type in both leaves and mature seeds (Fig. 4D, E). The flavonol profiles in mature seeds of these lines were qualitatively the same as that of the wild-type (see Supplementary Fig. S3 at *JXB* online). In addition, the accumulation of anthocyanins in leaves of over-expression lines did not appear to be affected (see Supplementary Fig. S4 at *JXB* online). In conclusion, over-expression of the *CHIL* gene in the *chil-1* mutant background could partially rescue the *chil* mutant phenotype.

To investigate whether higher over-expression of the *CHIL* gene could further impact flavonoid accumulation in wild-type *Arabidopsis*, transgenic lines over-expressing the *CHIL* gene in the Col background were generated and their flavonoid levels were analysed. The *CHIL* over-expression lines exhibited the same intense dark blue-purple DMACA staining compared with that of the wild-type (Fig. 4B), and the extractable and non-extractable PA contents were increased compared with Col (Fig. 4C, 30% and 40% increase in line 1). Kaempferol levels increased in the leaves and seeds of the over-expression lines compared with Col control plants (Fig. 4D, E). However, there was no change in the anthocyanin levels although the *CHIL* gene was highly expressed (see Supplementary Fig. S4 at *JXB* online). Taken together, these results indicate that over-expression of *CHIL* enhances the accumulation of both flavonol (kaempferol) and PAs in *Arabidopsis*.

### Expression levels of other flavonoid pathway genes in the *chil-1* mutant

**Fig. 5.**
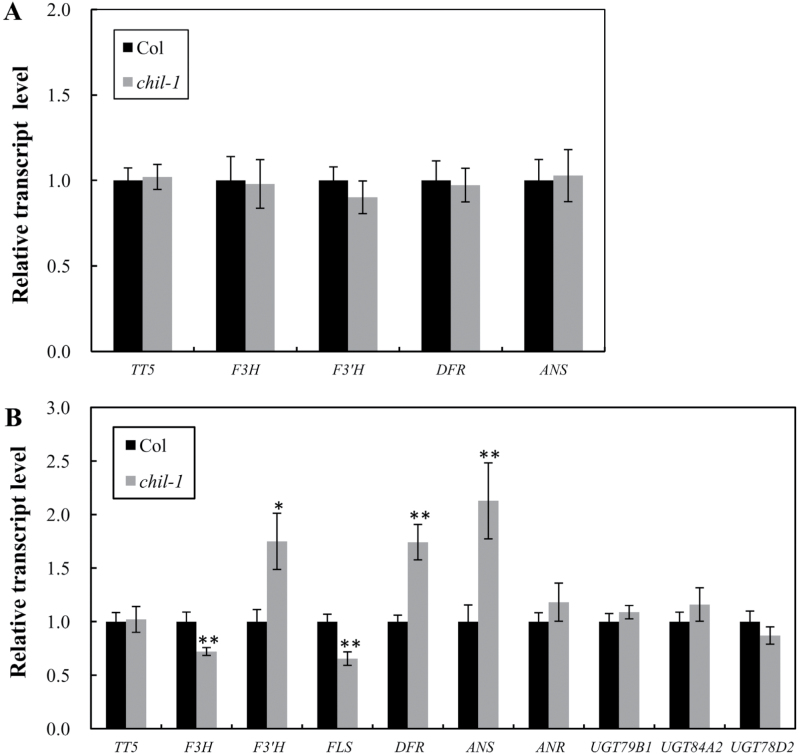
Relative transcript levels of flavonoid pathway genes in the *chil-1* mutant compared with the wild-type Col control. (A) Relative transcript levels of *TT5*, *F3H*, *F3′H*, *DFR*, and *ANS* in the 17-d-old seedlings of Col and *chil-1* mutant detected by qRT-PCR analysis. (B) Relative transcript levels of *TT5*, *F3H*, *F3′H*, *FLS*, *DFR, ANS*, *ANR*, and three *UGTs* in 4-d-old developing seed detected by qRT-PCR analysis. Data are presented as mean ±SD, Student’s *t* test (*n*=3, *P <0.05, **P <0.01).

To investigate whether the transcript levels of other pathway genes were affected by the loss-of-function of *CHIL* in *Arabidopsis*, qRT-PCR was performed to determine the transcript levels of key flavonoid pathway genes in both seedlings and developing seed tissues. The transcript levels of the *TT5*, *F3H*, *F3′H*, *DFR*, and *ANS* genes were almost unchanged in 17-d-old seedlings of the *chil-1* mutant, compared with the wild-type (Fig. 5A), consistent with the unaffected anthocyanin levels. In the developing seeds (4 d after flowering), none of the above genes changed by more than 2-fold in the *chil-1* mutant, although there were slight changes in the transcript levels of *F3′H*, *DFR*, and *ANS* (Fig. 5B). Taken together, the data suggest that CHIL does not function as a transcription factor in the flavonoid pathway.

### Neither loss- nor gain-of-function of *CHIL* affects the *tt5* and *chs* mutant phenotypes

In previous biochemical studies, CHIL could not be shown to have catalytic activity *in vitro* (Ngaki *et al.*, 2012). To confirm this further, the recombinant CHIL protein in *E. coli* was purified to high purity (see Supplementary Fig. S5A at *JXB* online) and its *in vitro* catalytic activity was tested with naringenin chalcone as the substrate. As reported previously, no activity was detected in this assay. Interestingly, when run on a non-denaturing PAGE gel, the recombinant CHIL protein displayed several bands with multiple integer sizes, indicating that CHIL can form different multimers (see Supplementary Fig. S5B at *JXB* online). By contrast, recombinant TT5 protein only appeared to form a tetramer (see Supplementary Fig. S5C at *JXB* online).

**Fig. 6.**
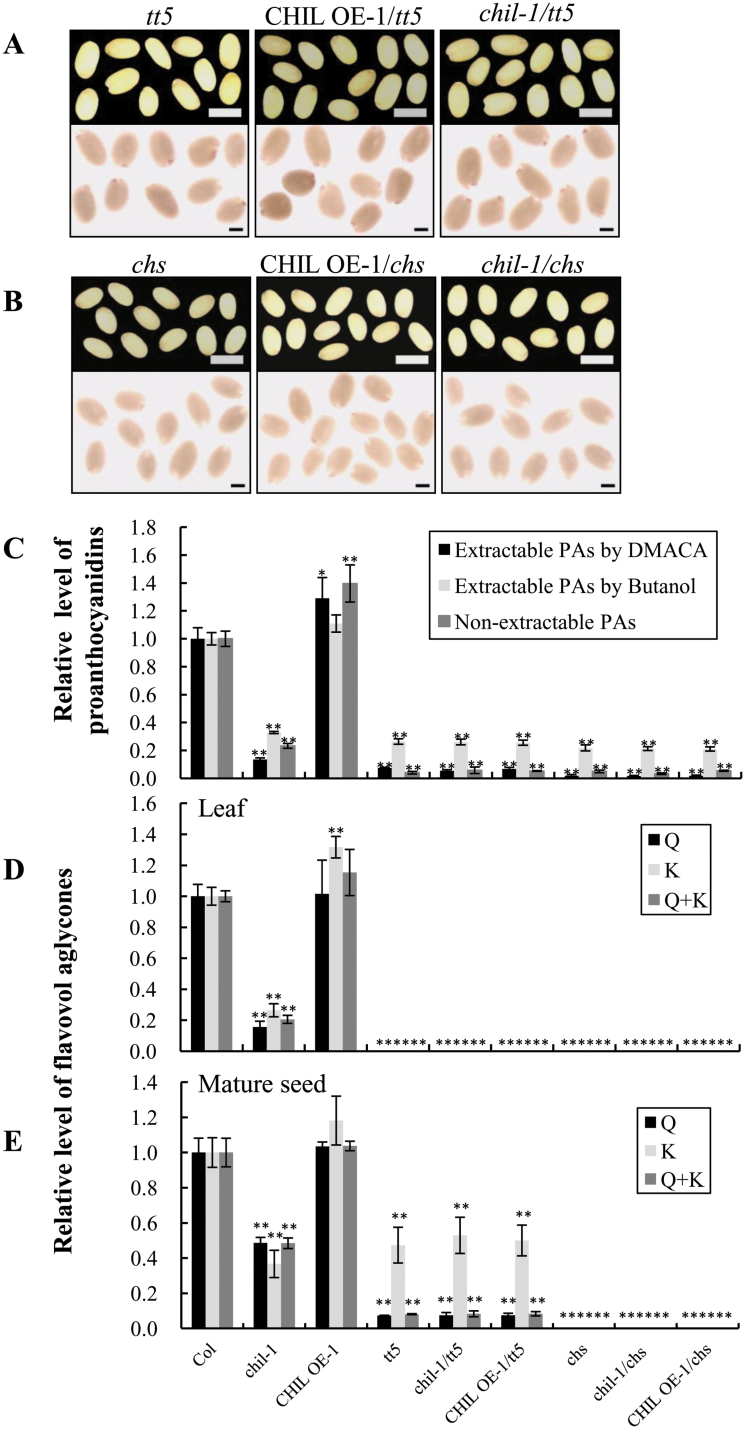
Characteristics of *CHIL* knockout and over-expression in the *tt5* and *chs* background. (A) Seed phenotype of the lines *tt5*, *chil-1*/*tt5*, CHIL OE-1/*tt5* (upper), stained with DMACA (lower). Bars=0.5mm. (B) Seed phenotype of *chs*, *chil-1*/*chs*, CHIL OE-1/*chs* (upper), stained with DMACA (lower). Bars=0.5mm. (C) Relative levels of proanthocyanidins in mature seeds of the above-mentioned plants. The extractable PAs measured by DMACA (2.64mg epicatechin equivalence g^–1^) and by butanol (1.08mg B2 equivalence g^–1^); non-extractable PAs (2.50mg B2 equivalence g^–1^) in Col were set as a value of 1.0, respectively. (D, E) Relative levels of the major flavonols [quercetin (Q) and kaempferol (K) aglycones] in 21-d-old seedlings (D) and mature seeds (E) of the above-mentioned plants. Levels of quercetin (4.79 µg g^–1^ dry weight and 584.27 µg g^–1^ dry weight in seedlings and seeds, respectively) and kaempferol (4.90 µg g^–1^ dry weight and 11.38 µg g^–1^ dry weight in seedlings and seeds, respectively) from wild-type control Col were set as a value of 1.0. (C, D, E) Data are presented as mean ±SD, Student’s *t* test (*n*=3, *P <0.05, **P <0.01). (This figure is available in colour at *JXB* online.)

To determine if CHIL has isomerase activity under *in vivo* conditions, an over-expressing line (CHIL OE-1) was crossed with the *tt5* mutant to investigate whether *CHIL* could rescue the *tt5* mutant phenotype. The CHIL OE-1/*tt5* line produced seeds with a *transparent testa* phenotype (as in the *tt5* mutant), and the seeds were not stained blue by DMACA, indicating that no PAs were produced in the CHIL OE-1/*tt5* line (Fig. 6A, upper panel). The flavonol profile of the *tt5* mutant on HPLC is similar to that of the *chil-1* mutants, except for the absence of quercetin and kaempferol in the *tt5* mutant (see Supplementary Figs S1 and S6 at *JXB* online). The PA and flavonol contents, and flavonol profiles, in CHIL OE-1/*tt5* were the same as those of the *tt5* mutant (Fig. 6C–E; see Supplementary Fig. S6 at *JXB* online). In addition, the anthocyanin levels did not differ between the CHIL OE-1/*tt5* and *tt5* seedlings (see Supplementary Fig. S7 at *JXB* online). Taken together, these results strongly indicated that *CHIL* cannot rescue the *tt5* phenotype and that *CHIL* does not encode an enzyme with CHI activity *in vivo*.

To investigate the genetic position of *CHIL* in the flavonoid pathway further, the *chil-1* mutant was next crossed with the *tt5* mutant. The *chil*/*tt5* double mutant produced progeny with a *tt* seed phenotype (Fig. 6, upper panel), and the levels of PA, flavonol, and anthocyanin, as well as the flavonol profiles, were not changed compared with the *tt5* mutant (Fig. 6C–E; see Supplementary Figs 5 and 6 at *JXB* online), indicating that CHIL does not function in the same way as TT5 in the flavonoid pathway.

Similarly, the *chil-1* mutant was also crossed with a *chs* mutant to produce the *chil*/*chs* double mutant, and the *CHIL* gene was also over-expressed in the *chs* mutant background by crossing the *chs* mutant with the CHIL OE-1 line. Both *chil*/*chs* double mutants and CHIL OE-1/*chs* lines produced seeds with *transparent testa* phenotypes, as in the *chs* mutant. Moreover, over-expression of the *CHIL* gene in the *chs* background could not rescue the *tt* phenotype, as measured by DMACA staining (Fig. 6B). In addition, both the double mutant and the *CHIL* over-expressing lines showed no difference in the accumulation levels of PAs, flavonols, and anthocyanins, as well as the flavonol profiles, compared with the *chs* mutant (Fig. 6B-D; see Supplementary Figs 7 and 8 at *JXB* online). All of these results indicate that *CHIL* does not function as a *CHS* in the flavonoid pathway.

### Temporal and spatial pattern of *CHIL* expression

**Fig. 7.**
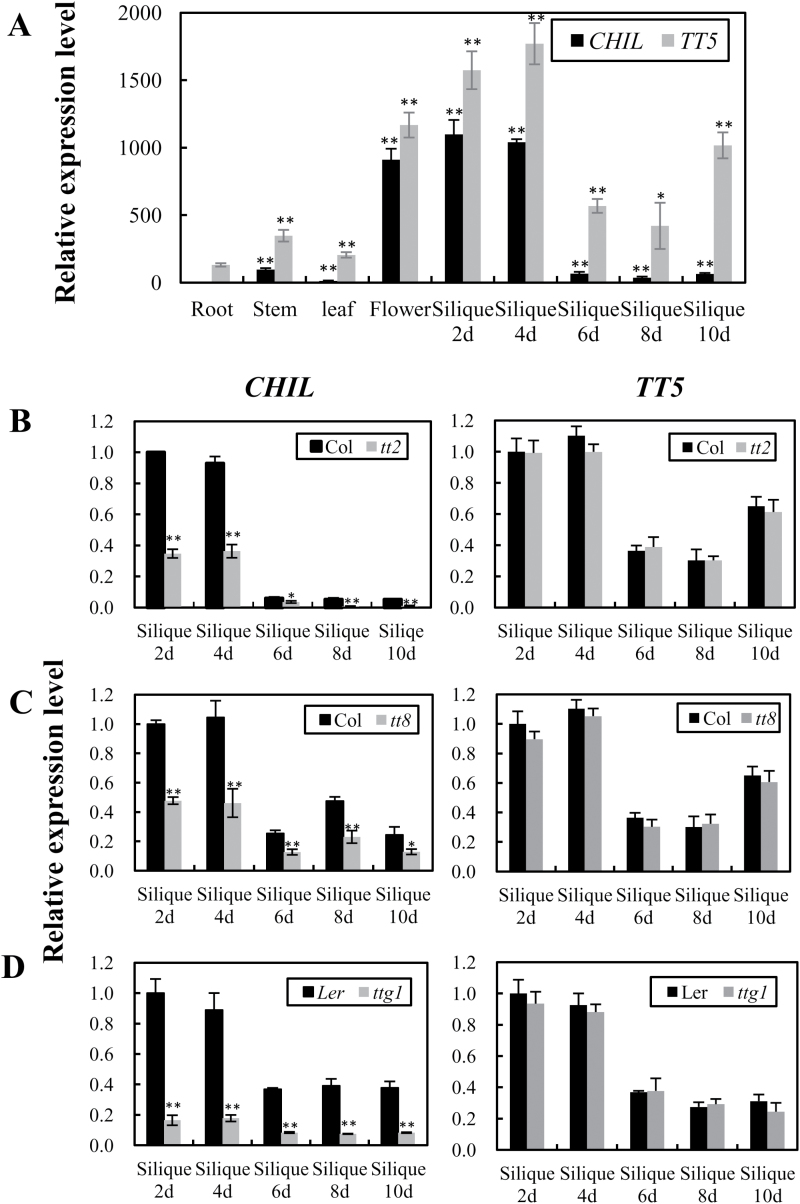
Expression pattern of *CHIL* and *TT5* genes in various tissues and mutants. (A) Relative transcript levels of *CHIL* and *TT5* in root, stem, rosette leaf, inflorescence, 2-, 4-, 6-, 8-, and 10-d-old siliques of wild-type *Arabidopsis* Col. (B–D) Relative transcript levels of the *CHIL* gene in developing seeds (represented by siliques) of *tt2* (B), *tt8* (C), and *ttg1* (D) mutants compared with the corresponding wild-type control. Data are presented as mean ±SD, Student’s *t* test (*n*=3, *P <0.05, **P <0.01).

To evaluate the expression pattern of the *CHIL* gene in *Arabidopsis*, its transcript levels were analysed in various organs and seed developmental stages using qRT-PCR. The *CHIL* gene was highly expressed in inflorescences and expressed at relatively lower levels in stems and rosette leaves. *CHIL* transcripts were hardly detectable in roots (Fig. 7A). Since *CHIL* affects the accumulation of flavonoids in seeds, *CHIL* transcript levels were also evaluated during different seed developmental stages (as represented by siliques). *CHIL* transcripts were at their highest level during the early stages of seed development (days 2 and 4), followed by a dramatic reduction at the later stages (days 6 and 10, Fig. 7A). The expression of the *TT5* gene showed a similar expression pattern in various organs and seed developmental stages (Fig. 7A). Nevertheless, the transcript pattern of *CHIL* is similar to the accumulation pattern of extractable PAs and a quercetin-rhamnoside-hexoside during seed development as previously reported by Routaboul *et al.* (2006), suggesting that *CHIL* is primarily involved in the biosynthesis of PAs and flavonols.

As it is known that the TT2/TT8/TTG1 transcription factor complex regulates the biosynthesis of PAs in the developing seeds of *Arabidopsis* (Baudry *et al.*, 2004), it was also evaluated whether *CHIL* was regulated by *TT2*, *TT8* or *TTG1* in the corresponding mutants. The *CHIL* transcript level was greatly reduced in the *tt2*, *tt8*, and *ttg1* mutants at all of the seed developmental stages compared with the wild-type (Fig. 7B-D). The reduction was particularly pronounced at the early stages of seed development (days 2 and 4), indicating that *CHIL* expression is regulated by *TT2*, *TT8*, and *TTG1* in the developing *Arabidopsis* seed.

Next, the 1742bp intergenic region of At5g05260 and At5g05270 (*CHIL*) was cloned and analysed using the Database of Plant Cis-acting Regulatory DNA Elements (PLACE, http://www.dna.affrc.go.jp/PLACE/) (Higo *et al.*, 1999) (see Supplementary Fig. S9 at *JXB* online). The S000144 element (CANNTG) is recognized by a transcription factor that controls the light-responsive and tissue-specific activation of phenylpropanoid biosynthesis genes (Hartmann *et al.*, 2005), and this element is present in several positions in the promoters of both *CHIL* and the *Arabidopsis ANR* gene (Debeaujon *et al.*, 2003). Another element, S000176 (CNGTTR), identified in the *Petunia hybrida CHSJ* gene is recognized by regulators of flavonoid biosynthesis (Solano *et al.*, 1995) and this element is also present in the promoters of *CHIL* and *ANR* (Debeaujon *et al.*, 2003). Analysis of the promoter region of the *TT5* gene revealed that it also has these two elements (ten for S000144 and three for S000176) that are shared by *CHIL* and *ANR* (see Supplementary Fig. S10 at *JXB* online), implying these genes may be co-ordinately regulated.

To examine the location of *CHIL* gene expression further, its promoter was fused to the *gusA* gene. This construct was introduced into *Arabidopsis* and various tissues were examined histochemically (see Supplementary Fig. S11 at *JXB* online). Staining of the *CHIL* promoter:GUS transgenic *Arabidopsis* plants with 5-bromo-4-chloro-3-indolyl-β-d-glucuronic acid revealed *CHIL* expression in young seedlings, the leaves of mature plants, the inflorescences, the peduncle of mature siliques, and immature seeds (before 6 d; see Supplementary Fig. S11 at *JXB* online). The expression pattern of *CHIL* detected by GUS staining was consistent with its transcript level pattern measured by qRT-PCR during seed development.

### CHIL is co-localized in the endoplasmic reticulum and physically interacts with TT5

**Fig. 8.**
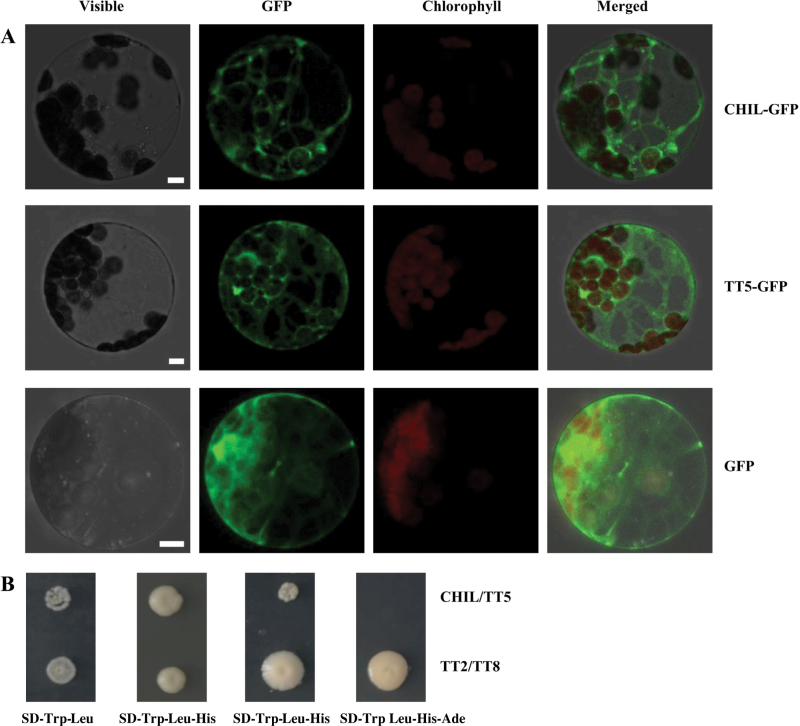
Co-localization and interaction of CHIL with TT5. (A) Subcellular localization of CHIL and TT5 by GFP assays in *Arabidopsis* protoplasts. Fluorescence signals were visualized using confocal laser scanning microscopy. From left to right: bright field, the green fluorescence, autofluorescence of the chloroplast, and merged images of CHIL-GFP fusion protein (upper panel), TT5-GFP fusion protein (middle panel), and GFP (lower panel). Bars=5 µm. (B) Interaction between CHIL and TT5 in yeast two hybrid assays. The yeast cells grew on the medium SD-Trp-Leu, SD-Trp-Leu-His, SD-Trp-Leu-His, and SD-Trp Leu-His-Ade. The interaction between TT2 and TT8 served as a positive control. (This figure is available in colour at *JXB* online.)

TT5 was reported to be localized to the endoplasmic reticulum and to act as an ER-membrane anchor for a metabolon involved in flavonoid biosynthesis (Saslowsky and Winkel-Shirley, 2001). To determine whether CHIL is localized to the endoplasmic reticulum, CHIL together with TT5 were fused to green fluorescent protein (GFP) for transient expression experiments using *Arabidopsis* leaf protoplasts (Fig. 8). Clear fluorescence signals for CHIL:GFP were detected in the endoplasmic reticulum (Fig. 8A); the same signal pattern was observed for TT5:GFP (Fig. 8B). The fluorescence signal patterns of CHIL:GFP and TT5:GFP were clearly distinct from that of control GFP (Fig. 8C). These results indicate that CHIL is localized to the endoplasmic reticulum as well as TT5 for flavonoid production.

The co-expression and co-localization of CHIL and TT5 suggest that the CHIL protein possibly interacts with TT5. In order to verify this hypothesis, a yeast two hybrid approach was taken to assess the CHIL and TT5 interaction, and TT2 and TT8 were used as positive controls. Yeast co-transformed with BD-CHIL and AD-TT5, BD-TT2 and AD-TT8, respectively, were both able to grow in the absence of Trp, Leu, and His, indicating that CHIL could indeed physically interact with TT5. However, CHIL/TT5 was not able to grow in the absence of Ade in contrast to TT2/TT8, indicating that the interaction of CHIL with TT5 was not as strong as that of TT2 with TT8 (Fig. 8D).

## Discussion

### CHIL is a functional gene involved in flavonoid biosynthesis in *Arabidopsis*

The biosynthesis of flavonoids has been well established in *Arabidopsis.* This was mainly achieved through the screening and characterization of a number of *transparent testa* mutants. In comparison with the dark brown seed colour in the wild-type, these *tt* mutants exhibit a pale yellow seed colour due to deficiencies in oxidized flavonoid compounds (mainly PAs), (Shirley *et al.*, 1995; Routaboul *et al.*, 2006; Appelhagen *et al.*, 2014). Nearly all of the *tt* mutants have been characterized *via* biochemical and/or genetic approaches and they have been shown to have mutations in structural, transporter or regulatory genes related to flavonoid production (Shirley *et al.*, 1992; Dixon *et al.*, 2005; Appelhagen *et al.*, 2014).

Research on PA biosynthesis is currently one of the hot spots in flavonoid metabolism research. Such research has advanced our understanding of the modification and transport of PA precursors in the pathway (Pang *et al.*, 2008; Zhao and Dixon, 2009; Zhao *et al.*, 2010), but there are still many unanswered questions regarding their biosynthetic mechanism. To explore further the candidate genes responsible for PA biosynthesis in *Arabidopsis*, a global gene expression analysis was performed in a *tt2* mutant that is deficient in PA biosynthesis in the seed coat. It was found that the *CHIL* gene was down-regulated in the *tt2* mutant, suggesting a potential role for CHIL in PA biosynthesis. The two null mutant alleles (*chil-1* and *chil-2*) produced dark brown seeds, as in the wild- type (Fig. 2A), a finding in sharp contrast to many other *tt* mutants with pale coloured seed phenotypes. Obviously, from the point view of the seed phenotype, the *CHIL* gene is different from all of the other known flavonoid pathway genes.

Several *tt* mutants that affect the loci specific for seed coat pigmentation are deficient in anthocyanins in the leaves, including *tt4, tt5, tt6*, and *tt7*, but the anthocyanin accumulation in the *chil* mutants was not affected in the leaves, another finding that was obviously distinct from these *tt* mutants (Appelhagen *et al.*, 2014). The accumulation levels of anthocyanins in the two *chil* mutants were similar to those of the wild-type, so CHIL is not involved in anthocyanin production. Unlike other flavonoid pathway genes, the *CHIL* gene may act in flavonoid biosynthesis *via* an as yet uncharacterized mechanism.

Further analysis showed that the seeds of the two *chil* mutant lines accumulated markedly reduced levels of PAs and flavonols when compared with the wild-type (Figs 1, 2), but the phenotype of the *chil* mutant could be recovered *via* complementation (Fig. 4). This observation unambiguously shows that CHIL is involved in flavonoid biosynthesis and plays a major role in PA biosynthesis in *Arabidopsis*.

Both CHIL and TT5 belong to the CHI superfamily of *Arabidopsis* (Ngaki *et al.*, 2012), and a strong association of *CHIL* expression levels with the expression levels of *TT5* was observed in this study and elsewhere (Ma, 2002; Oravecz *et al.*, 2006; Yonekura-Sakakibara *et al.*, 2008; Pan *et al.*, 2009). However, the *CHIL* gene could not rescue the deficiency of flavonoid accumulation and failed to recover the seed colour phenotypes of the *tt5* mutant (Fig. 6). Therefore, the *CHIL* gene is not a redundant *TT5* gene, but rather a new functional gene in the flavonoid biosynthetic pathway, particularly in the PA branch.

### CHIL functions with TT5 for enhancing flavonoid accumulation in *Arabidopsis*

**Fig. 9.**
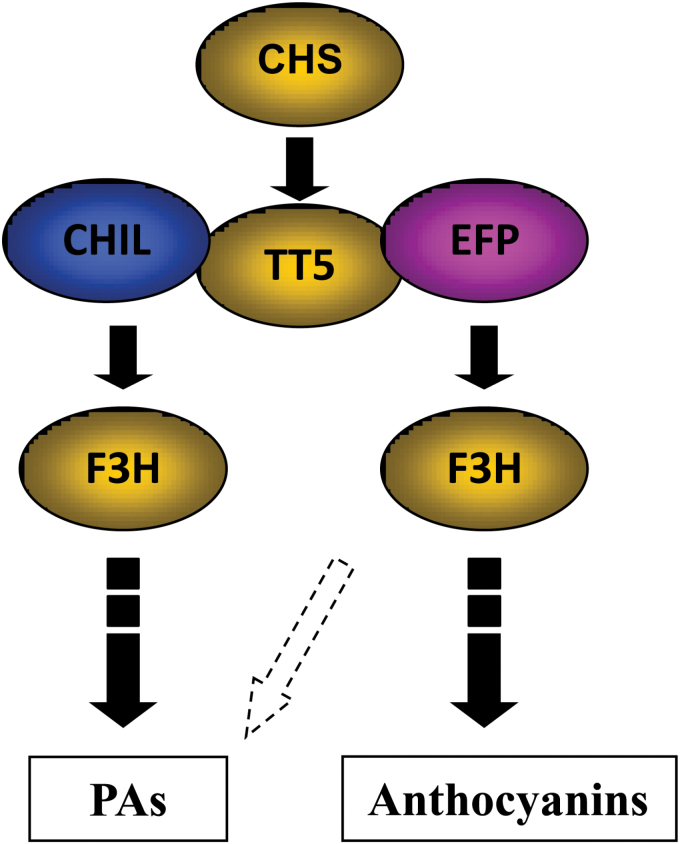
Hypothetical model illustrating the role of CHIL and EFP proteins in flavonoid biosynthesis. CHIL and EFP proteins are downstream of CHS, and interact with TT5 in the pathway for proanthocyanidin and anthocyanin biosynthesis, respectively. They may function as anchor, enhancer or chaperone for specific flavonoid accumulation. (This figure is available in colour at *JXB* online.)

Generally, loss-of-function mutations in genes at different steps in the same pathway produce different metabolite profiles (Yonekura-Sakakibara *et al.*, 2008). In the present study, it was found that *chil* mutants displayed a similar flavonol profile to *tt5* (see Supplementary Figs S1 and S6 at *JXB* online), the profile of which is largely distinct from those of the *chs* and *f3h* mutants (see Supplementary Figs S8 and S12 at *JXB* online). These results suggest that CHIL most probably acts at the same step as TT5 and that it is located immediately downstream of CHS and upstream of F3H (Fig. 9).

Apart from a strong association of *CHIL* expression with that of *TT5*, *CHIL* and *TT5* are also co-expressed in various organs and seed developmental stages, which is consistent with the presence of two *cis*-acting elements shared by *CHIL* and *TT5* in their promoter regions (Fig. 7; see Supplementary Fig. S11 at *JXB* online). In addition, both CHIL and TT5 are localized to the endoplasmic reticulum, potentially associated with a metabolon for flavonoid synthesis (Winkel-Shirley *et al*., 2001).

Most importantly, CHIL was observed to interact physically with TT5 in yeast two-hybrid assays. X-ray crystallography analyses of the CHI-related proteins revealed that the structure of CHIL is much more similar to TT5 than to those of the other CHI-related proteins (Jez *et al.*, 2000; Ngaki *et al.*, 2012). CHIL may thus be a more suitable partner for heterodimerization with TT5 than any of the other CHI-related proteins. Taken together, these lines of evidence suggest that CHIL may conceivably act as a partner for TT5 and may thus enhance TT5 catalytic activity.

### A unique role of the CHIL proteins in flavonoid biosynthesis

Unfortunately, no catalytic activity has been detected for recombinant CHIL protein in this or previous studies (Jez *et al.*, 2000; Ngaki *et al.*, 2012). As with the CHIL protein, no catalytic activity with naringenin chalcone was observed for recombinant EFP (Enhance Flavonoid Production) protein, a homologue of CHIL in *Ipomoea nil* (Japanese morning glory) (Morita *et al.*, 2014). Therefore, the CHIL proteins (CHIL and EFP) do not seem to have catalytic activity with naringenin chalcone, although they have enzyme-like structures (Morita *et al.*, 2014).

In addition, expression analysis showed that the transcript levels of several investigated structural genes of the flavonoid pathway were not affected (namely *TT5*, *ANR* and three *UGTs*), or did not change by more than 2.1-fold (namely *F3H*, *F3′H*, *FLS*, *DFR*, and *ANS*) in the *chil-1* mutant. Similar results were observed for *EFP*, as deficiency of the *EFP* gene did not affect the transcript levels of the other pathway genes (*CHS*, *DFR*, and *3GT*) in the *efp* mutant (Morita *et al.*, 2014). Thus, CHIL does not appear to function as a transcription factor in flavonoid biosynthesis.

It is possible that the contrast between the pale-coloured flowers of the *efp* mutant and the colourless flowers of the *chi* mutant in *I. nil* plant, was due to differential accumulation levels of anthocyanins (Iida *et al.*, 2004; Morita *et al.*, 2014), a situation similar to the differential accumulation levels of PAs and flavonols in the *Arabidopsis chil* and *tt5* mutants. The transcript levels of both *CHIL* and *EFP* genes were completely absent in the corresponding mutants (Morita *et al.*, 2014), indicating that they are not weak alleles of *CHIL* genes. On the other hand, loss-of-function of *CHIL* in *Arabidopsis* is different from loss-of-function of *EFP* of *I. nil* in that it affected the accumulation of PAs and flavonols rather than anthocyanins. It is possible that there may be another EFP orthologue in *Arabidopsis* that is involved in anthocyanin accumulation (Fig. 9).

As hypothesized by Morita *et al.* (2014), the CHIL proteins may function in guiding the proper folding of polyketide intermediates into homo- or hetero-multimeric proteins with TT5. Alternatively, CHIL may function as an anchor, chaperone or enhancer to promote the accumulation of flavonoids by stabilizing or enhancing the activity of the corresponding CHI proteins (Fig. 9). Although the detailed molecular mechanism remains to be elucidated of how CHIL and EFP promote flavonoid production, they appear to be unique positive regulator proteins that lack catalytic activities. It remains to be seen whether these hypothesized regulation mechanisms underlying CHIL and EFP are universal in plants.

## Supplementary data

Supplementary data can be found at *JXB* online.

Supplementary Fig. S1. Representative HPLC chromatograms of the *chil-1* mutants and Col.

Supplementary Fig. S2. Over-expression of the *CHIL* gene in the *chil-1* mutant and Col background.

Supplementary Fig. S3. Representative HPLC chromatograms of the over-expression lines in the *chil-1* mutant and Col backgrounds.

Supplementary Fig. S4. Anthocyanin accumulation in the *CHIL* over-expression lines in the *chil* and Col backgrounds.

Supplementary Fig. S5. Purified recombinant CHIL and TT5 protein on denaturing and native PAGE.

Supplementary Fig. S6. Representative HPLC chromatograms of *CHIL* loss-of-function and over- expression lines in the *tt5* background.

Supplementary Fig. S7. Anthocyanin accumulation in the *CHIL* loss-of-function and over-expression lines in the *tt5* and *chs* mutant backgrounds.

Supplementary Fig. S8. Representative HPLC chromatograms of *CHIL* loss-of-function and over-expression lines in the *chs* background.

Supplementary Fig. S9. *Cis*-acting elements upstream the ORF of the *CHIL* gene as predicted by PLACE.

Supplementary Fig. S10. *Cis*-acting elements upstream the ORF of the *TT5* gene as predicted by PLACE.

Supplementary Fig. S11. Tissue-specific promoter activity of the *CHIL* promoter fused with GUS.

Supplementary Fig. S12. Representative HPLC chromatograms of *F3H* loss-of-function compared with the *chil-1* mutant in the Col background.

Supplementary Table S1. The probe sets with expression more than 2 fold down-regulated in seeds of the *tt2* mutant of *Arabidopsis*.

Supplementary Table S2. The primer sequences used in the present study.

Supplementary Data

## References

[CIT0001] Alonso JM Stepanova AN Leisse TJ 2003 Genome-wide insertional mutagenesis of *Arabidopsis thaliana* . Science 301, 653–657. 12893945 10.1126/science.1086391

[CIT0002] Appelhagen I Thiedig K Nordholt N Schmidt N Huep G Sagasser M Weisshaar B 2014 Update on transparent testa mutants from *Arabidopsis thaliana*: characterisation of new alleles from an isogenic collection. Planta 240, 955–970. 24903359 10.1007/s00425-014-2088-0

[CIT0003] Baudry A Heim MA Dubreucq B Caboche M Weisshaar B Lepiniec L 2004 TT2, TT8, and TTG1 synergistically specify the expression of *BANYULS* and proanthocyanidin biosynthesis in *Arabidopsis thaliana* . The Plant Journal 39, 366–380. 15255866 10.1111/j.1365-313X.2004.02138.x

[CIT0004] Clough SJ Bent AF 1998 Floral dip: a simplified method for *Agrobacterium*-mediated transformation of *Arabidopsis thaliana* . The Plant Joural 16, 735–743. 10.1046/j.1365-313x.1998.00343.x 10069079

[CIT0005] Debeaujon I Nesi N Perez P Devic M Grandjean O Caboche M Lepiniec L 2003 Proanthocyanidin-accumulating cells in *Arabidopsis* testa: regulation of differentiation and role in seed development. The Plant Cell 15, 2514–2531. 14555692 10.1105/tpc.014043 PMC280558

[CIT0006] Dixon RA Xie DY Sharma SB 2005 Proanthocyanidins: a final frontier in flavonoid research? New Phytologist 165, 9–28. 15720617 10.1111/j.1469-8137.2004.01217.x

[CIT0007] Ferreyra FML Rius SP Casati P 2012 Flavonoids: biosynthesis, biological functions, and biotechnological applications. Frontiers in Plant Science 3, 222. 23060891 10.3389/fpls.2012.00222 PMC3460232

[CIT0008] Hartmann U Sagasser M Mehrtens F Stracke R Weisshaar B 2005 Differential combinatorial interactions of *cis*-acting elements recognized by R2R3-MYB, BZIP, and BHLH factors control light-responsive and tissue-specific activation of phenylpropanoid biosynthesis genes. Plant Molecular Bio*logy* 57, 155–171. 15821875 10.1007/s11103-004-6910-0

[CIT0009] Hassan S Mathesius U 2012 The role of flavonoids in root–rhizosphere signalling: opportunities and challenges for improving plant–microbe interactions. Journal of Experimental Bota*ny* 63, 3429–3444. 22213816 10.1093/jxb/err430

[CIT0010] He X Blount JW Ge S Tang Y Dixon RA 2011 A genomic approach to isoflavone biosynthesis in kudzu (*Pueraria lobata*). Planta 233, 843–855. 21221632 10.1007/s00425-010-1344-1

[CIT0011] Higo K Ugawa Y Iwamoto M Korenaga T 1999 Plant *cis*-acting regulatory DNA elements (PLACE) database: 1999. Nucleic Acids Research 27, 297–300. 9847208 10.1093/nar/27.1.297 PMC148163

[CIT0012] Iida S Morita Y Choi JD Park KI Hoshino A 2004 Genetics and epigenetics in flower pigmentation associated with transposable elements in morning glories. Advances in Biophysics 38, 141–159. 15493332

[CIT0013] Jez JM Bowman ME Dixon RA Noel JP 2000 Structure and mechanism of the evolutionarily unique plant enzyme chalcone isomerase. Nature Structural and Molecular Biology 7, 786–791. 10.1038/79025 10966651

[CIT0014] Jiang WB Huang HY Hu YW Zhu SW Wang ZY Lin WH 2013 Brassinosteroid regulates seed size and shape in *Arabidopsis* . Plant Physiology 162, 1965–1977. 23771896 10.1104/pp.113.217703 PMC3729775

[CIT0015] Jiang WB Yu DQ 2009 Arabidopsis WRKY2 transcription factor mediates seed germination and postgermination arrest of development by abscisic acid. BMC Plant Biology 9. 10.1186/1471-2229-9-96 PMC2719644 19622176

[CIT0016] Karimi M Inze D Depicker A 2002 GATEWAY vectors for *Agrobacterium*-mediated plant transformation. Trends in Plant Science 7, 193–195. 11992820 10.1016/s1360-1385(02)02251-3

[CIT0017] Koornneef M 1981 The complex syndrome of *ttg* mutants. *Arabidopsis* Information Service 18, 45–51.

[CIT0018] Kozlowska A Szostak-Wegierek D 2014 Flavonoids: food sources and health benefits. Roczniki Państwowego Zakładu Higieny 65, 79–85. 25272572

[CIT0019] Lattanzio V Lattanzio VMT Cardinali A 2006 Role of phenolics in the resistance mechanisms of plants against fungal pathogens and insects. Phytochemistry: Advances in Research 23–67.

[CIT0020] Lee ER Kang GH Cho SG 2007 Effect of flavonoids on human health: old subjects but new challenges. Recent Patents in Biotechnology 1, 139–150. 10.2174/187220807780809445 19075837

[CIT0021] Lepiniec L Debeaujon I Routaboul JM Baudry A Pourcel L Nesi N Caboche M 2006 Genetics and biochemistry of seed flavonoids. Annual Review of Plant Biology 57, 405–430. 10.1146/annurev.arplant.57.032905.105252 16669768

[CIT0022] Li Y Tanner G Larkin P 1996 The DMACA-HC protocol and the threshold proanthocyanidin content for bloat safety in forage legumes. Journal of the Science of Food and Agriculture 70, 89–101.

[CIT0023] Ma L 2002 Genomic evidence for COP1 as a repressor of light-regulated gene expression and development in *Arabidopsis* . The Plant Cell 14, 2383–2398. 12368493 10.1105/tpc.004416 PMC151224

[CIT0024] Morita Y Takagi K Fukuchi-Mizutani M 2014 A chalcone isomerase-like protein enhances flavonoid production and flower pigmentation. The Plant Journal 78, 294–304. 24517863 10.1111/tpj.12469

[CIT0025] Nesi N Jond C Debeaujon I Caboche M Lepiniec L 2001 The *Arabidopsis TT2* gene encodes an R2R3 MYB domain protein that acts as a key determinant for proanthocyanidin accumulation in developing seed. The Plant Cell 13, 2099–2114. 11549766 10.1105/TPC.010098 PMC139454

[CIT0026] Ngaki MN Louie GV Philippe RN Manning G Pojer F Bowman ME Li L Larsen E Wurtele ES Noel JP 2012 Evolution of the chalcone-isomerase fold from fatty-acid binding to stereospecific catalysis. Nature 485, 530–533. 22622584 10.1038/nature11009 PMC3880581

[CIT0027] Oravecz A Baumann A Mate Z Brzezinska A Molinier J Oakeley EJ Adam E Schafer E Nagy F Ulm R 2006 CONSTITUTIVELY PHOTOMORPHOGENIC1 is required for the UV-B response in *Arabidopsis* . The Plant Cell 18, 1975–1990. 16829591 10.1105/tpc.105.040097 PMC1533968

[CIT0028] Pan Y Michael TP Hudson ME Kay SA Chory J Schuler MA 2009 Cytochrome P450 monooxygenases as reporters for circadian-regulated pathways. Plant Physiology 150, 858–878. 19386812 10.1104/pp.108.130757 PMC2689971

[CIT0029] Pang Y Peel GJ Sharma SB Tang Y Dixon RA 2008 A transcript profiling approach reveals an epicatechin-specific glucosyltransferase expressed in the seed coat of *Medicago truncatula* . Proceedings of the National Academy of Sciences, USA 105, 14210–14215. 10.1073/pnas.0805954105 PMC2544603 18772380

[CIT0030] Pang Y Peel GJ Wright E Wang Z Dixon RA 2007 Early steps in proanthocyanidin biosynthesis in the model legume *Medicago truncatula* . Plant Physiology 145, 601–615. 17885080 10.1104/pp.107.107326 PMC2048810

[CIT0031] Pang Y Wenger JP Saathoff K 2009 A WD40 repeat protein from *Medicago truncatula* is necessary for tissue-specific anthocyanin and proanthocyanidin biosynthesis but not for trichome development. Plant Physiology 151, 1114–1129. 19710231 10.1104/pp.109.144022 PMC2773055

[CIT0032] Ralston L Subramanian S Matsuno M Yu O 2005 Partial reconstruction of flavonoid and isoflavonoid biosynthesis in yeast using soybean type I and type II chalcone isomerases. Plant Physiology 137, 1375–1388. 15778463 10.1104/pp.104.054502 PMC1088328

[CIT0033] Rosso MG Li Y Strizhov N Reiss B Dekker K Weisshaar B 2003 An *Arabidopsis thaliana* T-DNA mutagenized population (GABI-Kat) for flanking sequence tag-based reverse genetics. Plant Molecular Biology 53, 247–259. 14756321 10.1023/B:PLAN.0000009297.37235.4a

[CIT0034] Routaboul JM Kerhoas L Debeaujon I Pourcel L Caboche M Einhorn J Lepiniec L 2006 Flavonoid diversity and biosynthesis in seed of *Arabidopsis thaliana* . Planta 224, 96–107. 16395586 10.1007/s00425-005-0197-5

[CIT0035] Saito K Yonekura-Sakakibara K Nakabayashi R Higashi Y Yamazaki M Tohge T Fernie AR 2013 The flavonoid biosynthetic pathway in *Arabidopsis*: structural and genetic diversity. Plant Physiology and Biochemistry 72, 21–34. 23473981 10.1016/j.plaphy.2013.02.001

[CIT0036] Saslowsky D Winkel-Shirley B 2001 Localization of flavonoid enzymes in *Arabidopsis* roots. The Plant Journal 27, 37–48. 11489181 10.1046/j.1365-313x.2001.01073.x

[CIT0037] Sheen J 2002 A transient expression assay using *Arabidopsis* mesophyll protoplasts http://genetics.mgh.harvard.edu/sheenweb/.

[CIT0038] Shimada N Aoki T Sato S Nakamura Y Tabata S Ayabe S 2003 A cluster of genes encodes the two types of chalcone isomerase involved in the biosynthesis of general flavonoids and legume-specific 5-deoxy(iso)flavonoids in *Lotus japonicus* . Plant Physiology 131, 941–951. 12644647 10.1104/pp.004820 PMC166860

[CIT0039] Shirley BW Hanley S Goodman HM 1992 Effects of ionizing radiation on a plant genome: analysis of two *Arabidopsis* transparent testa mutations. The Plant Cell 4, 333–347. 1354004 10.1105/tpc.4.3.333 PMC160133

[CIT0040] Shirley BW Kubasek WL Storz G Bruggemann E Koornneef M Ausubel FM Goodman HM 1995 Analysis of *Arabidopsis* mutants deficient in flavonoid biosynthesis. The Plant Journal 8, 659–671. 8528278 10.1046/j.1365-313x.1995.08050659.x

[CIT0041] Solano R Nieto C Avila J Canas L Diaz I Paz-Ares J 1995 Dual DNA binding specificity of a petal epidermis-specific MYB transcription factor (MYB.Ph3) from *Petunia hybrida* . The EMBO Journal 14, 1773–1784. 7737128 10.1002/j.1460-2075.1995.tb07166.x PMC398271

[CIT0042] Winkel-Shirley B 2001 Flavonoid biosynthesis. A colorful model for genetics, biochemistry, cell biology, and biotechnology. Plant Physiology 126, 485–493. 11402179 10.1104/pp.126.2.485 PMC1540115

[CIT0043] Wisman E Hartmann U Sagasser M Baumann E Palme K Hahlbrock K Saedler H Weisshaar B 1998 Knock-out mutants from an En-1 mutagenized *Arabidopsis thaliana* population generate phenylpropanoid biosynthesis phenotypes. Proceedings of the National Academy of Sciences, USA 95, 12432–12437. 10.1073/pnas.95.21.12432 PMC22848 9770503

[CIT0044] Xie DY Sharma SB Paiva NL Ferreira D Dixon RA 2003 Role of anthocyanidin reductase, encoded by BANYULS in plant flavonoid biosynthesis. Science 299, 396–399. 12532018 10.1126/science.1078540

[CIT0045] Yao LH Jiang YM Shi J Tomas-Barberan FA Datta N Singanusong R Chen SS 2004 Flavonoids in food and their health benefits. Plant Foods for Human Nutrition 59, 113–122. 15678717 10.1007/s11130-004-0049-7

[CIT0046] Yonekura-Sakakibara K Fukushima A Nakabayashi R 2012 Two glycosyltransferases involved in anthocyanin modification delineated by transcriptome independent component analysis in *Arabidopsis thaliana* . The Plant Journal 69, 154–167. 21899608 10.1111/j.1365-313X.2011.04779.x PMC3507004

[CIT0047] Yonekura-Sakakibara K Tohge T Matsuda F Nakabayashi R Takayama H Niida R Watanabe-Takahashi A Inoue E Saito K 2008 Comprehensive flavonol profiling and transcriptome coexpression analysis leading to decoding gene-metabolite correlations in *Arabidopsis* . The Plant Cell 20, 2160–2176. 18757557 10.1105/tpc.108.058040 PMC2553606

[CIT0048] Zhang F Maeder ML Unger-Wallace E 2010 High frequency targeted mutagenesis in *Arabidopsis thaliana* using zinc finger nucleases. Proceedings of the National Academy of Sciences, USA 107, 12028–12033. 10.1073/pnas.0914991107 PMC2900673 20508152

[CIT0049] Zhao J Dixon RA 2009 MATE transporters facilitate vacuolar uptake of epicatechin 3′-*O*-glucoside for proanthocyanidin biosynthesis in *Medicago truncatula* and *Arabidopsis* . The Plant Cell 21, 2323–2340. 19684242 10.1105/tpc.109.067819 PMC2751950

[CIT0050] Zhao J Pang Y Dixon RA 2010 The mysteries of proanthocyanidin transport and polymerization. Plant Physiology 153, 437–443. 20388668 10.1104/pp.110.155432 PMC2879784

