# Supplementary material for: Role of a chalcone isomerase-like protein in flavonoid biosynthesis in Arabidopsis thaliana
Source: J Exp Bot. 2015 Sep 7;66(22):7165–79. doi: 10.1093/jxb/erv413 (PMC4765788; doi:10.1093/jxb/erv413)
Supplement: Supplementary Data [file supp_66_22_7165__index.html]

Role of a chalcone isomerase-like protein in flavonoid biosynthesis in Arabidopsis thaliana — Role of a chalcone isomerase-like protein in flavonoid biosynthesis in Arabidopsis thaliana — Role of a chalcone isomerase-like protein in flavonoid biosynthesis in Arabidopsis thaliana — Supplementary Data 

# Role of a chalcone isomerase-like protein in flavonoid biosynthesis in *Arabidopsis thaliana*

## Supplementary Data

Data files

- Supplementary Data - Supplementary Data
