# Supplementary material for: Role of a chalcone isomerase-like protein in flavonoid biosynthesis in Arabidopsis thaliana
Source: J Exp Bot. 2015 Sep 7;66(22):7165–79. doi: 10.1093/jxb/erv413 (PMC4765788; doi:10.1093/jxb/erv413)
Supplement: Supplementary Data [file supp_erv413_jexbot150128_file001.pdf]

Supporting Information

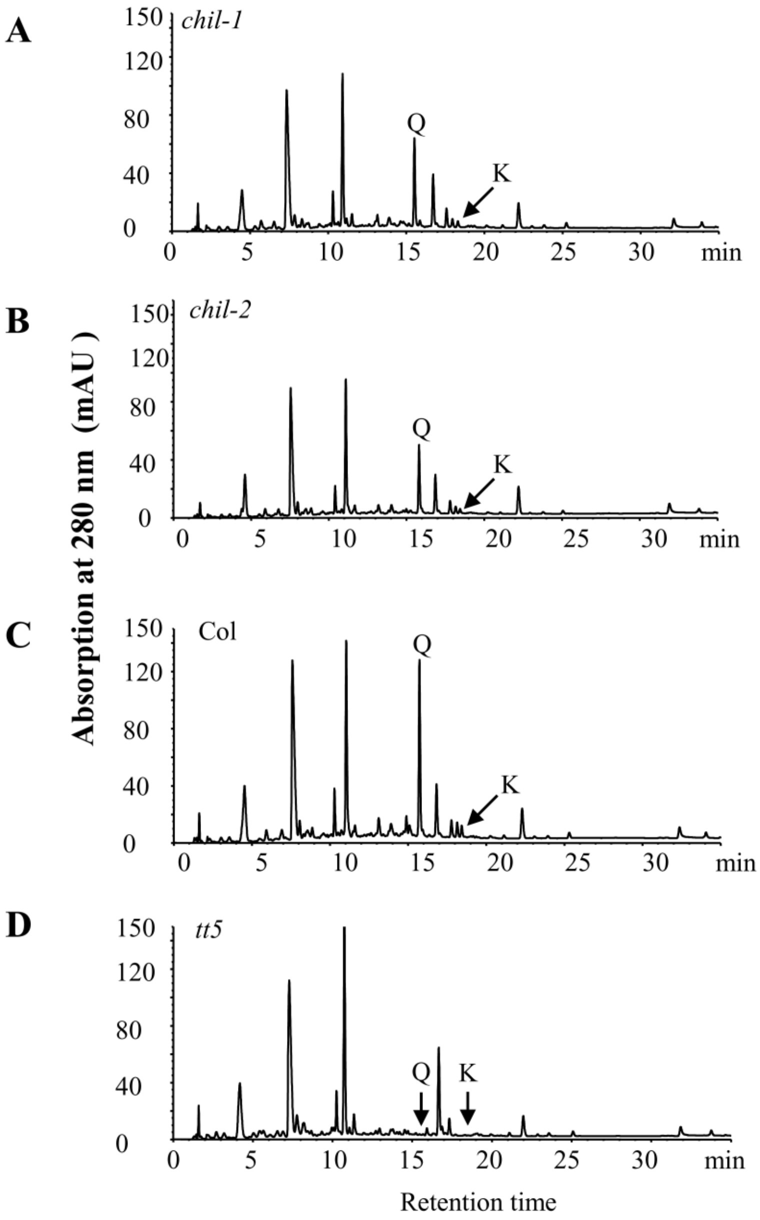

Figure S1. Representative HPLC chromatograms of the *chil-1* mutants and Col. The chromatograms were obtained from methanolic extracts of mature seeds of *chil-1* (**A**), *chil-1* (**B**), Col control (**C**) and *tt5* (D).

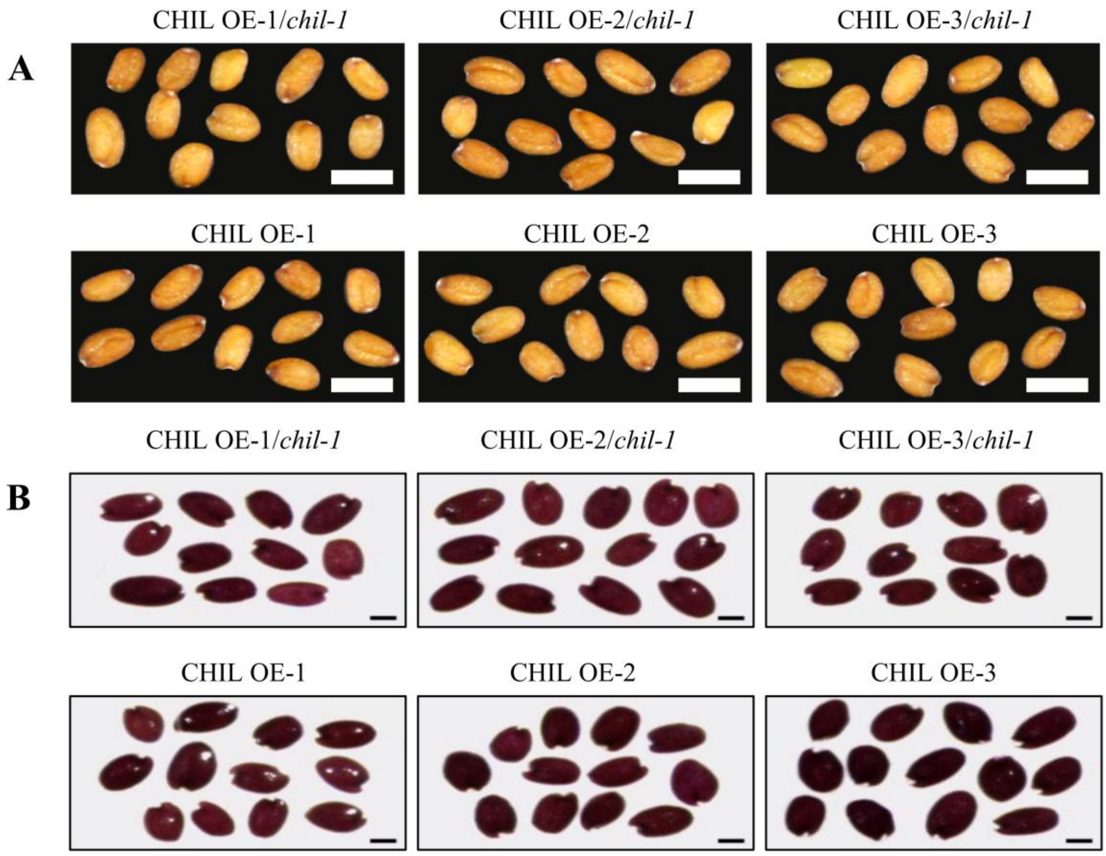

Figure S2. Over-expression of *CHIL* gene in the *chil-1* mutant and Col background.

(A) Seed phenotype of the CHIL OE-1/*chil-1*, CHIL OE-2/*chil-1*, CHIL OE-3/*chil-1*, CHIL OE-1, CHIL OE-2 and CHIL OE-3 before staining with DMACA. Bars=0.5 mm.

(B) Seed phenotype of the above mentioned mutant lines after staining with DMACA for 20 days. Bars=0.2 mm.

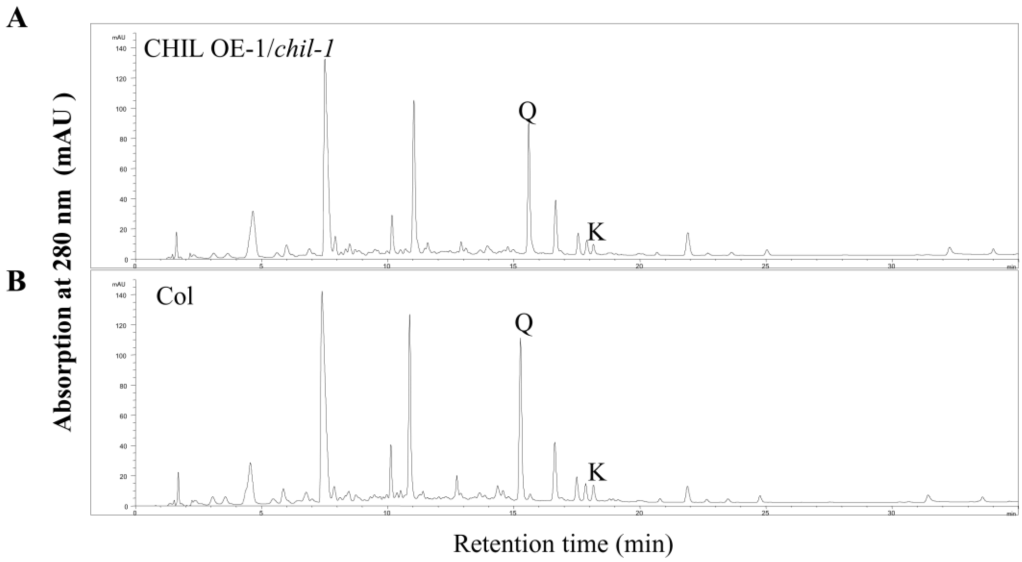

Figure S3. Representative HPLC chromatograms of the over-expression lines in the *chil-1* mutant and Col background.

The chromatograms were obtained from methanolic extracts of mature seeds of CHIL OE-1/*chil-1* (A) and wild type control Col (B). The over-expression of *CHIL* gene could complement the *chil* mutant phenotype in flavonol accumulation.

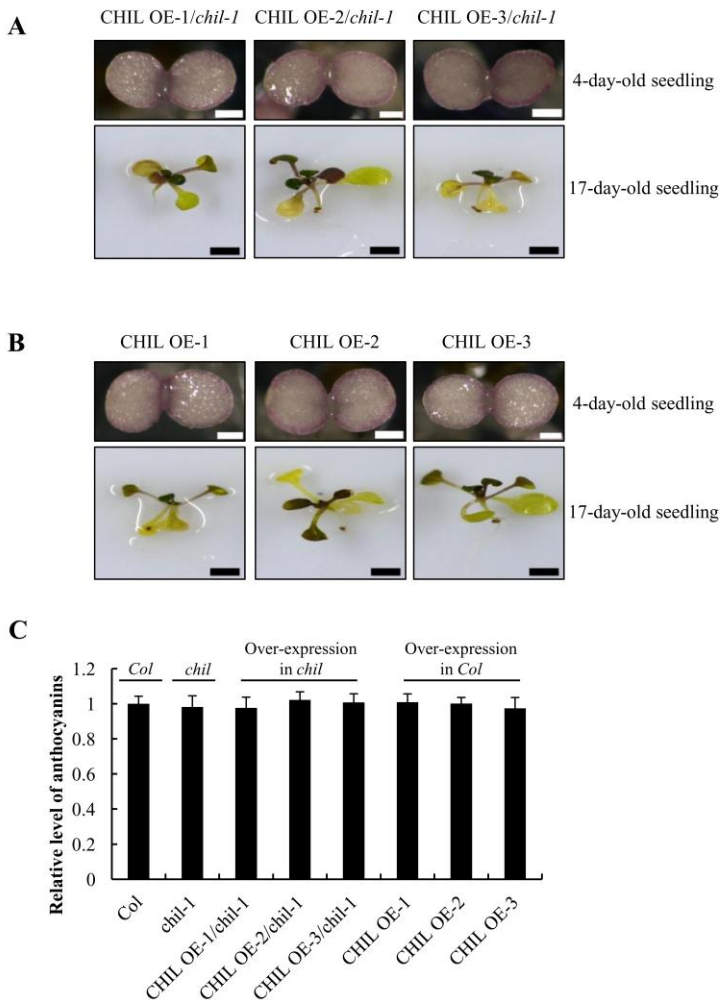

Figure S4. Anthocyanin accumulation in the *CHIL* over-expression lines in the *chil* and Col background.

(A) Anthocyanin accumulation in 4-day-old seedlings of *Arabidopsis* (the upper panels, bars=0.5 mm) and 17-day-old seedlings (the bottom panels, bars=5 mm) of CHIL OE-1/*chil-1*, CHIL OE-2/*chil-1* and CHIL OE-3/*chil-1*.

(B) Anthocyanin accumulation in the 4-day-old seedlings of *Arabidopsis* (the upper panels, bars=0.5 mm) and 17-day-old seedlings (the bottom panels, bars=5 mm) of CHIL OE-1, CHIL OE-2 and CHIL OE-3.

(C) Relative level of anthocyanins in the 17-day-old seedlings of Col, *chil-1*, CHIL OE-1/*chil-1*, CHIL OE-2/*chil-1*, CHIL OE-3/*chil-1*, CHIL OE-1, CHIL OE-2 and CHIL OE-3. The level of anthocyanins in wild-type control Col (1.57 mg/ g dry weight) was set as value of 1.0. Data are presented as mean ± SD, student's t-test (n=3，*P<0.05，**P<0.01).

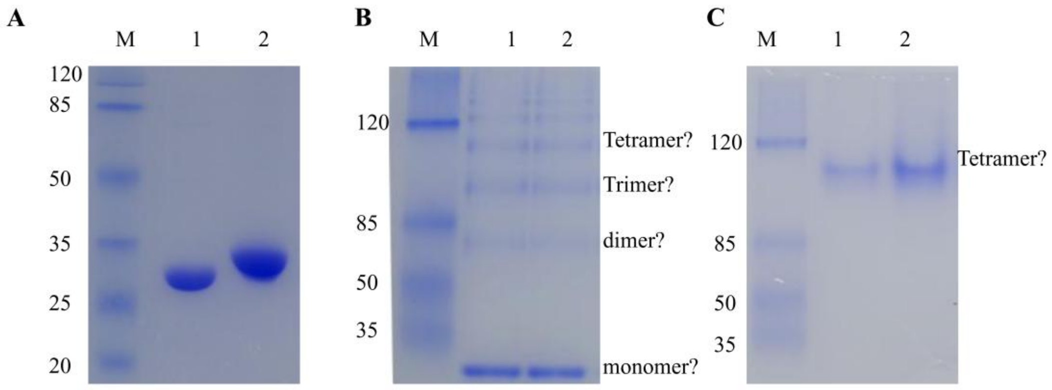

Figure S5. Purified recombinant CHIL and TT5 proteins on denaturing and native PAGE.

(A) Recombinant CHIL and TT5 proteins on denaturing SDS-PAGE. M, protein marker; 1, recombinant CHIL protein; 2, recombinant TT5 protein.

(B-C) Recombinant CHIL (B) and TT5 (C) proteins on native PAGE. M, protein marker;1-2, recombinant protein.

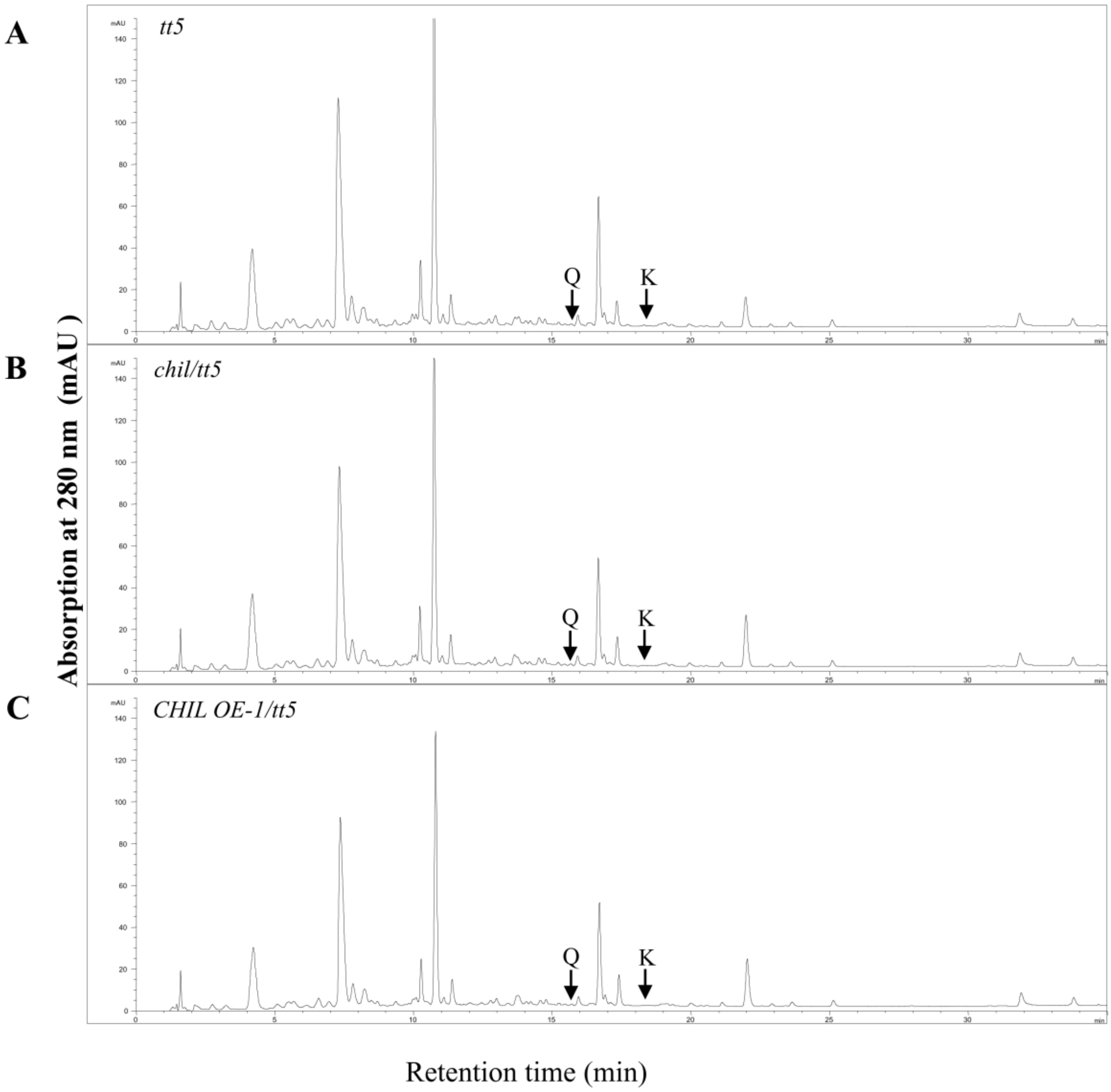

Figure S6. Representative HPLC chromatograms of *CHIL* loss-of-function and over- expression lines in the *tt5* background.

The chromatograms were obtained from methanolic extracts of mature seeds from lines of *tt5* (A), *chil-1*/*tt5* (B) and CHIL OE-1/*tt5* (C). All the lines showed the same flavonol profile on HPLC.

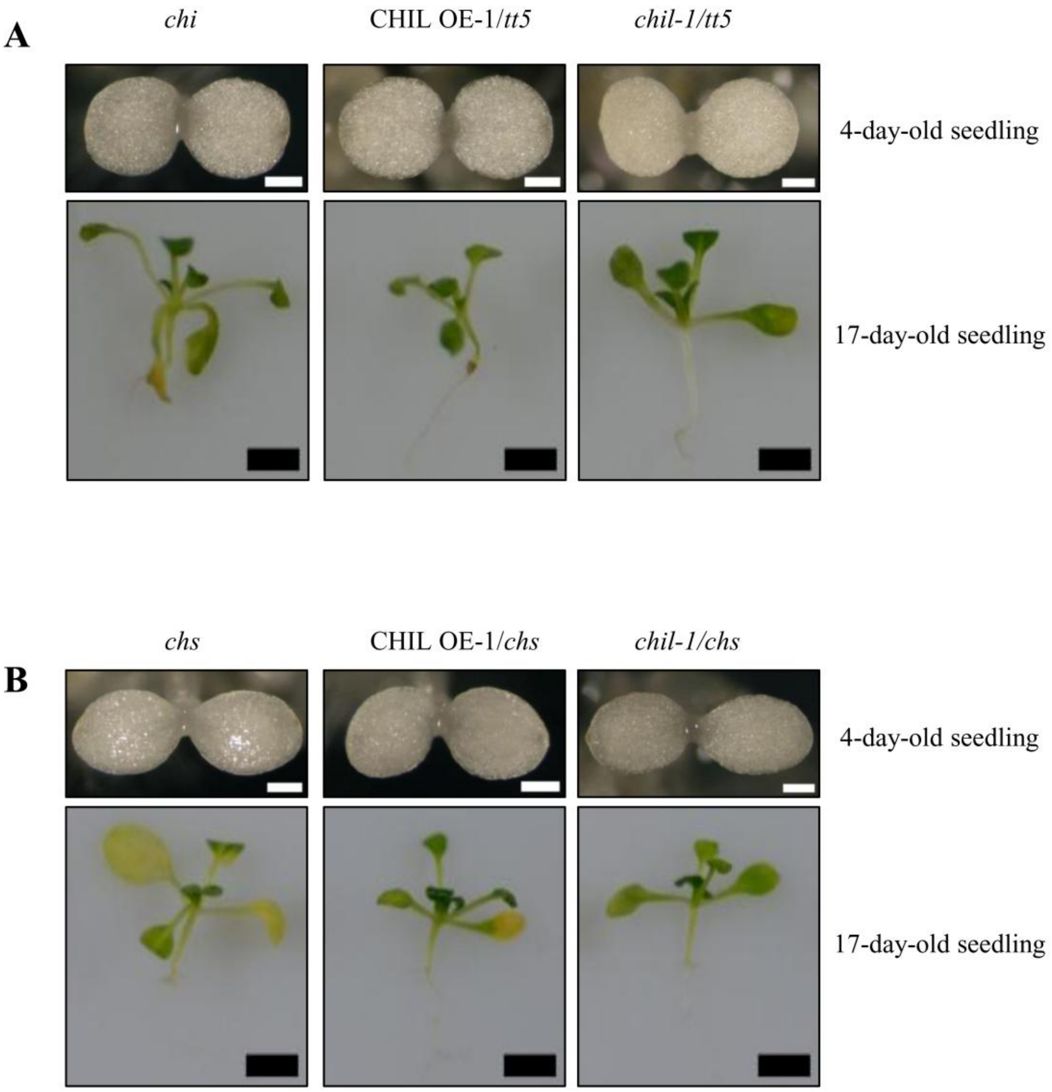

Figure S7. Anthocyanin accumulation in the *CHIL* loss-of-function and over-expression lines in the *tt5* and *chs* mutant background.

Anthocyanin accumulation in the 4-day-old seedlings of *Arabidopsis* (the upper panels, bars=0.5 mm) and 17-day-old seedlings (the bottom panels, bars=5 mm) of *tt5*, *chil-1*/*tt5* and CHIL OE-1/*tt5* (A); of *chs*, *chil-1*/*chs* and CHIL OE-1/*chs* (B).

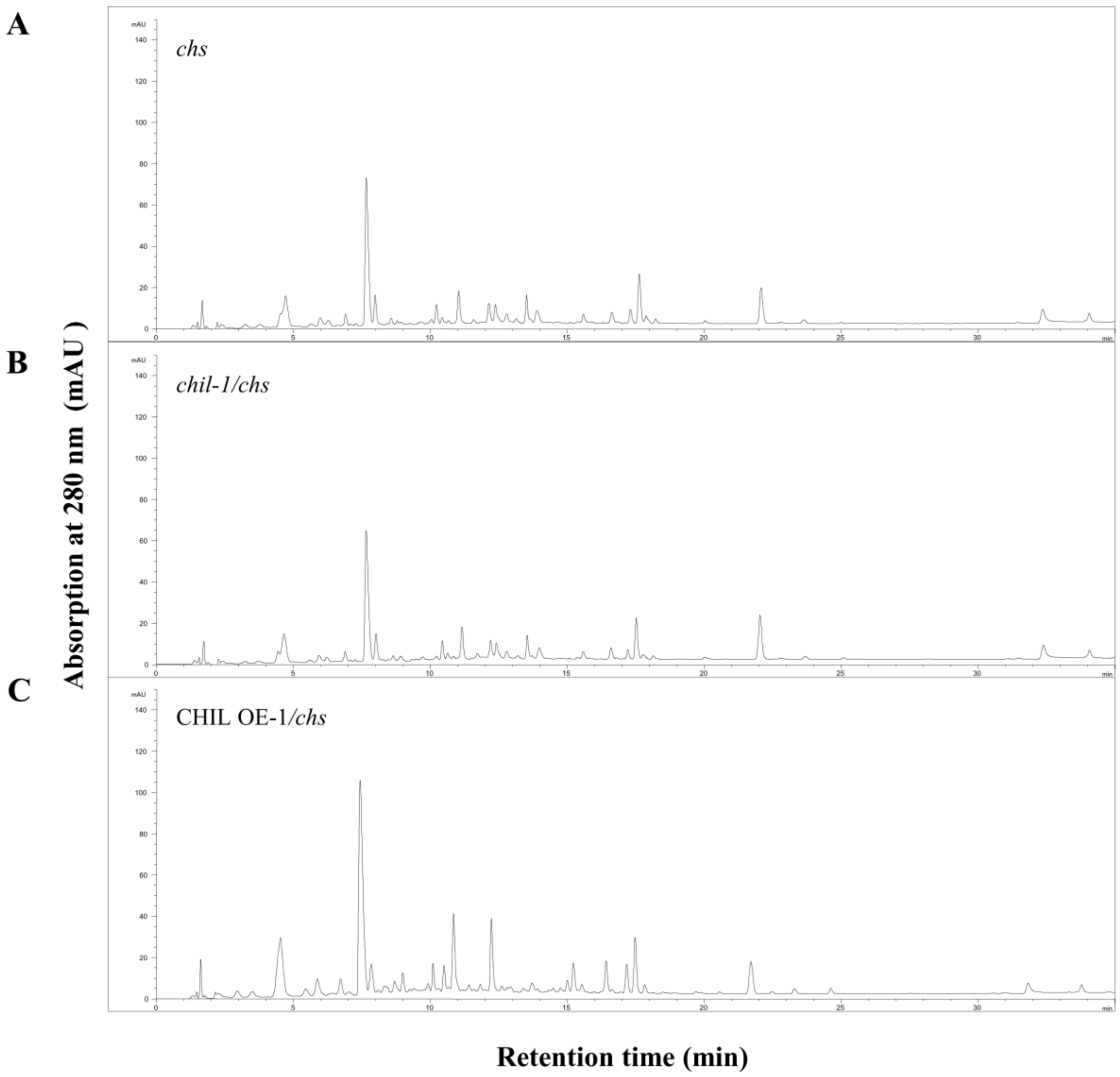

Figure S8. Representative HPLC chromatograms of *CHIL* loss-of-function and over-expression lines in the *chs* background.

The chromatograms were obtained from methanolic extracts of mature seeds from lines of *chs* (A), *chil-1*/*chs* (B) and CHIL OE-1/*chs* (C). All the lines showed the same flavonol profile on HPLC as *chs* mutant.

TGGGGTGGAGTCAAGCATTTTTTTTTTTAAATCTCAATTTGGAGAAATTATTTGAGTTCGAAAAGGTTTTTCTTATCT
ATGATGAGGAATAAGCTATTTATAGCCATGAAACAAATGCTTTTGTTTAAGTGTAATCTATGGAAAATAAATGTGCTTT
AAATGTTGAAGAAAACAATGTTAAATGAATCCACGATTTTTCAACAGCTCATATGTAAGACATGTTTTTTTCTTTGATT
ATTCCTTATCCCTTATAAATCTTGTGGATGTTAAATTGGTAATGCATCATTATGAAATCCTTGTCCCTTTTAGGTAGCGC
CATCATTAGAAAAACCACGATTTTTCAACAGCTCATATGTCTAATGTATTACATGTTGTGTTTTAAGTACATTTCTATTT
AATTACCAACTTTTTACTTTTCCAAATTCGTCTAAATCTAATCTCTAAGGATAAATTTGCTTACGATTCCTTAATCCTTA
TCCCTTTAAAATCTTGTTGATGTTAAATTGTTAATGCTGTTTATGAAATCTTTATCCCCTTTTAGGTAGCGCCACGTTAA
AAACAATATGGACATGGAGATTTGGGCTTATAAAAACTGTAAATATTTGGCCCACTTAAAAATTGATTCAGCTTCTGT
AAATTTAATCCTAGCAGCTTCCATTCGGCTCGACATGCGTAGCAAAAGGAATACCCACGACTATCCATCAACCATGA
AATTAATTAGGAGGCGAATTAAAACATAATCAAATTACATAAGGACTAATTAAGAAAAGCTAGAGATTGTGATTAGAG
AATAGAGATTATACCATGACTTGCTGAAGCCAATTTTTGGCACTTTGCACACATTACCCAATCTCTTTCAGTTGTCTCT
TCGATCCTTTGTTCCCACTTGGCTCTTAGTTTATGTGTCGAAAGATGGGCATAGCTTTCAATAGTCAGTTTCGATTTGC
AGCAGTAACACTTACTTACCAGTTCAAAAATGTGATAATGATCGCAATAGATTGAGATATGACTTATGAGTGATCCCC
AAACTCACGGCTTCGATTAGTCCTCGCTTCGATGCCATAAGCTGAGCCTCTAAACCCGTAATAGCGGAGTCATGAATT
GATCCTTTCATGATTTGGGAGGTCGAGATAGAAGAGAAGACGAAAAAGGGTTTTTATACGTTACTGCTTTAAAGACA
CTCTTTATTTCTCTTTTTTTTTTTTTTTTTTTTTTTTGGGTCTTTGTCATCATCAGTTTACTTTACGTTACTGATTTCAAG
AGCCGTTGTGGAGAAATGTGGCTGCTTCTAATTTTGTATCTAATTTATGTACGCTTTGTTTCACATTTGACATCTGATC
TCTTTTTACTGTATCATAGCAATTCTTTCTTCTATCTCTTTTAATTATTATACTTCTATACCTTCATTTGTTAGGTAGAATG
AGTGGTTGTGGACTGAGTGCTTGATCCAAACACGTGGCTTCCTGGTGGGTCGCTAACAAACAATTGAGATCACAAG
CTTTGGTAGCAAAAAAATTGAATTCATAAAAAGAATACGTCGTCTTTTAGATCACAAGCTTTGAACGTTAGCAAAATT
ATGTTACCTACCACCAGTCCCTGGCTTATTATATAGAGAGAGATATTCACATTTACAATTTCGATATTGTTTCTCTAAGA
TCGTAAAATAGTACACCAAGCCATTTATAAATTTCTTCACTCTCAGATTCAAAATAATATAAATTAGAAAGTAAGTCTT
ACACTC

Figure S9. *Cis*-acting elements upstream the ORF of the *CHIL* gene as predicted by PLACE.

The binding sites are highlighted in yellow (S000144: EBOXBNNAPA) and green (S000176: MYBCORE), respectively. The capital A in blue is the A for start codon ATG. The primer positions are boxed.

AGAACAATTGCTTAAGTAAAGCAAGACCATTACCCTCCTTCAAGGTTTCGCTATCTGCTCGTTCCTTTGTCTTGACTT
GGCCTCTGTTTTTCTGTTTCGTTTTCCTTACTTATTGCCTATTGGTGATAGTTCTGTTTCGTAGTGTTGCTCTCCTTTGT
TTGCTGTCTTGTCCCTTTGGGTGCAGTTCAAAAAATTAAAATCTAGGGGCTGCTGTTGAATGTTTTGCGAATTTTTCA
ACCATATAGTTATATCAATATTCAAGTATTGGTGATAGTTCTTTTCCACAATGGTCAGCAAAATAAAATATCAACAAAA
GTTCAACCACGAAGTTTAGTAGATATGAAATGAATATTGCGCATCCCACGTTGGAGATATAAGTTTTATGGTCCGGAC
TCCCTTACTTCATACTTCATACTTCATATATAGTATATTTTACTCAAAGAAAAAAAATGGACAAGTGAAAATCTAGTGA
ATTAATGTATTCACCTAAATTTGAGATATAATATTTTCTGGTGTGATAGACATTACTCACTATTCAATGACTCAAAATTT
AAAACATGCATGTCAGAACACAATTAAATTCGAACCAATTGAATACACATTTGAAATTAGTGTCCTTATAATTGAATT
TTGATGCGCAAGTCATTTTGATGAGAAAATTAGAAGATCAAAAATGGACAAATCAAGATACATGGAGAATTAGGATA
TGAAAAATAGTTTTAAAATTATTGTCTATATGTATGGAACATTAGAACATTTTGGTAGAGAGTAAATGATCAAAAAAAT
CTGGTTATCTGAGGTGTAAAAAGATTGGCTACTGCAATAAACATGGCACAGATGTTAGAAAATTTAAAGTTAAATGAT
TCAAAGTTTTATATAATTTGGGACAATTCAAAATCTTGTGACTTATATAATGTGATATTAATGTATTCACTAAACTACAA
AGTAAATAATTAATTTACTTTTAAATTTGGTAGAGGTTGATTTTGCAATAATGACTATTAAATGAATAAAGTTCAAACA
TATCACAGTAGACGTTTAATTTCGAACTACTTATGTATATAAATCGACAATACATTTAAAGTATTTAAATGCGCAGTTAA
ATTGAAGCCATAAAATTTGCAAACCGAAAATTTATGCTTTCAAACTGTAAACCATTTATGCTTTCACACTGATTAAAG
GGCCCTTCTATAAACTTTTCCATTAAATATTTTTCATTTAAATATTAGATGATCTTAAATGGTTTACTCAAAGTAAATATA
TATATATATGAAGAATTAAGAAAGTGTGGAGCATTTTAAAACTTTTTTTTCTCTAGTTTTTTTTGCTTTTTTTTTTGGGC
AACGTTCGTTCCCTCGAGTCTATTAAATTTTTTGCCATTTTTGTTTTTTGTTTTTTCAGTGACAAACTCACATGGATTT
CATTTCAGATTCAGTTAGATTTTATTATACTTCAGCCCAGTGTAGGAACAACCGGAACCCAAAATCTAGAGAGCATTC
ATGGTGGGGTTCAATAGCCCATGTAATAGCCCACTTCCCGGCATCAGAACTGACAAATCCAAAATTGAATTGAAATG
TGTTTATAACCCGATTAGTTTTAATATTACGGAACAAACAAATCAAAACCATAACTCAAACTAAACGAAGTCATATAA
TAGATATGATAAATTTATATTAAACGTTTTATTAAATATAAAAGAGTGTTAAAAGTTTTTATTTATCTAAAATGAACATA
GTTAAACGAAAACCCAACCAAATCTAAGTACAATTGTTCAAAACCAAAGTGAACCAAGTTGAATTATTGTTTTTAAT
TCACAAAACGATTTGTTGAAATAAGGGCTGAAGTCGTAGAAGCTTTAATAGATAAGAAAAGAAAGAGATATGTTGAT
ATTGAGAATACCCAAAAAACTAGGATTTCTGAATATTAGAAACTAGAGAACGTAAGAAATTCTTTGATTTTTTCTTTG
ATTTTCCGAAAGTAAGAATTAGAGAATACTTTTTCTGAAAAGCTATTTTTTTTTTTTTTTAATTTTATTGATCAATCAACT
GAATACTGAATACAAAAGAAAACCTGATTTATTTATAATAAAACTCAATTTATTTATAATATAATTCAAAAACCTAGATA
AACTCTAAAATAATCATATAATTATCTCAAATAACCAAAATATATAAAATCAATAATATGAAAGATAACGATAGAAAATA
TTTGAAATTATCCTAACTATGTGCTCTGCATATTTAAGGGCTCAAAGCTTCAACCACCAATTGTCAATGCATCTCCCAA
TCAAAATATAAAAAAAGAGACGAAAACGAAAGTACACGTGCTTACACATCCAACACTCGTAATCGTAACTATTGCTA
CCTACCCTTCTCTCTTCTACTTAACCCCAAAGGCCCCAAAAACACAATCAAGAAAAGCTCTGTAACATTATTATCAAT
CGAAATTCCAACCGACTCAACAATG

Figure S10. *Cis*-acting elements upstream the ORF of the *CHI* gene as predicted by PLACE.

The binding sites are highlighted in yellow (S000144: EBOXBNNAPA) and green (S000176: MYBCORE), respectively. The capital ATG in blue is start codon.

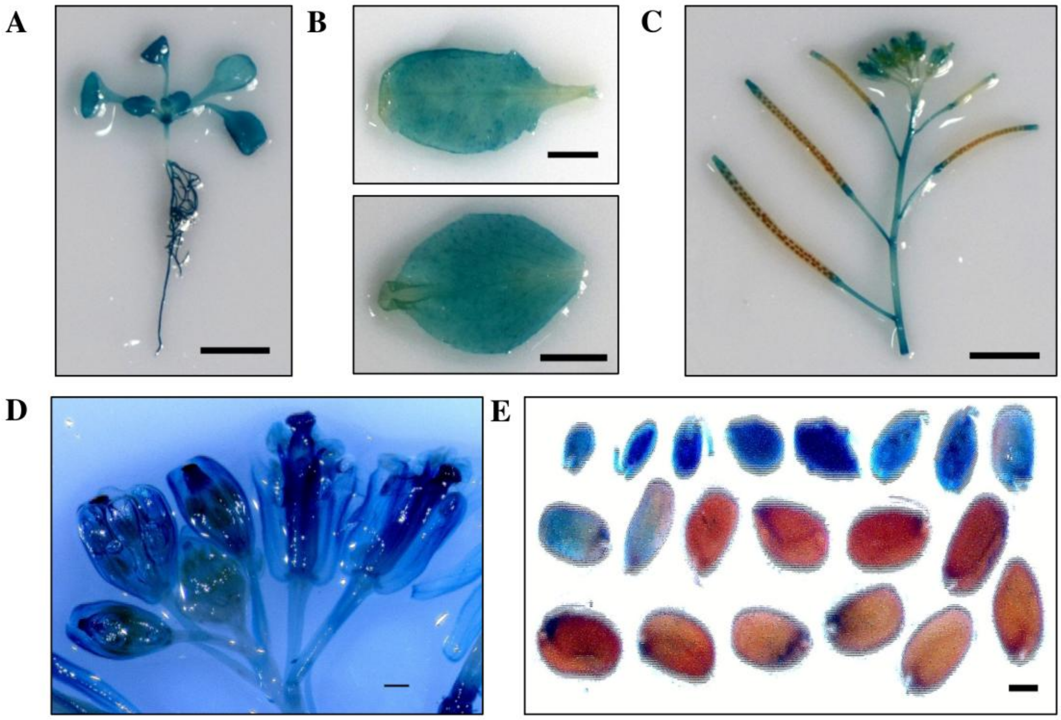

Figure S11. Tissue-specific promoter activity of *CHIL* promoter fused with GUS.

(A) GUS staining of 10-day-old seedlings of Arabidopsis.

(B) GUS staining of rosette leaves (the upper panels) and cauline leaves (the bottom panels).

(C, D) GUS staining of young siliques and inflorescences.

(E) GUS staining of young seeds (from 1- to 10-day-old seeds).

Bars=5 mm in A, B and C; Bars=0.5 mm in D; Bars=0.2 mm in E.

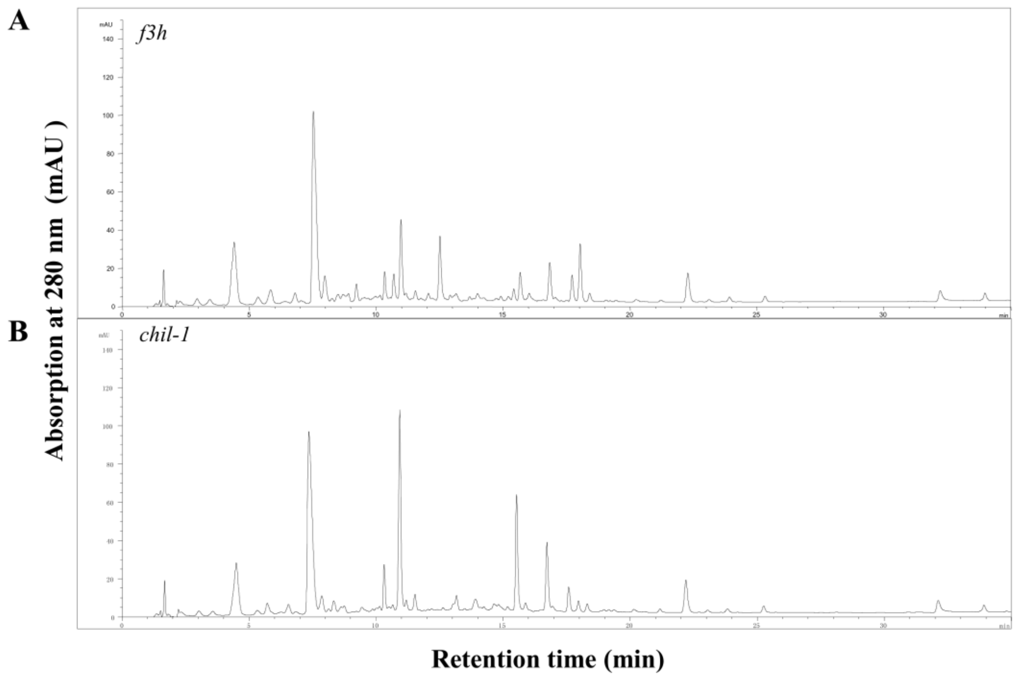

Figure S12. Representative HPLC chromatograms of *F3H* loss-of-function in comparison to *chil-1* mutant in the Col background.

The chromatograms were obtained from methanolic extracts of mature seeds from lines of *f3h* (A) and *chil-1* (B).

Table S1. The probe sets with expression more than 2 fold down-regulated in seeds of the tt2 mutant of Arabidopsis.

Probe Set ID	Representative Public ID	Target Description	Fold change (tt2/Ler)	Fold change (Ler/tt2)	Pts	Pta
266169_at	At2g38900	putative protease inhibitor	0.01	97.41	0.000109361	0
249215_at	At5g42800	dihydroflavonol 4-reductase	0.02	43.93	5.70715E-05	0
249700_at	At5g35530	40S ribosomal protein S3	0.03	39.53	3.93708E-05	0
264401_at	At1g61720	anthocyanidin reductase	0.03	34.82	0.000957021	0
262083_at	At1g56100	hypothetical protein predicted by genemark.hmm	0.04	23.60	6.43787E-05	0
254283_s_at	At4g22870	anthocyanidin synthase - like protein putative leucoanthocyanidin dioxygenase, Arabidopsis thaliana, PID:g1575699	0.05	22.03	0.00096712	0
249704_at	At5g35550	Myb-related transcription factor LBM2-like protein	0.07	14.07	8.06641E-05	0
250083_at	At5g17220	glutathione S-transferase-like protein	0.07	13.82	9.5222E-06	0
262119_s_at	At1g02930	glutathione S-transferase	0.07	13.70	1.01387E-05	0
263757_at	At2g21430	cysteine proteinase similar to cysteine proteinase RD19A (thiol protease)	0.09	11.30	2.33055E-05	0
245556_at	At4g15400	HSR201 like protein	0.12	8.08	2.53147E-05	0
248937_at	At5g45770	putative protein contains similarity to unknown protein (pir||T04868)	0.13	7.79	4.12587E-06	0
252306_at	At3g49270	hypothetical protein	0.13	7.79	5.14966E-05	0
256290_at	At3g12203	serine carboxypeptidase	0.13	7.74	0.000108163	0
253902_at	At4g27170	NWMU4 - 2S albumin 4 precursor	0.13	7.68	4.32825E-05	0
264740_at	At1g62070	unknown protein	0.13	7.44	3.82742E-06	0
249703_at	At5g35560	putative protein strong similarity to unknown protein (gb|AAD21756.1)	0.14	7.35	0.000129594	0
251202_at	At3g63040	putative protein predicted protein	0.14	7.27	6.48631E-06	0
264737_at	At1g62210	unknown protein	0.14	7.19	4.1869E-06	0
264079_at	At2g28490	putative seed storage protein (vicilin-like)	0.15	6.87	0.000356611	0
247424_at	At5g62850	LIM7 - like protein LIM7, Lilium longiflorum, EMBL:LLORF07	0.15	6.82	0.000324415	0
262431_at	At1g47540	trypsin inhibitor 2	0.15	6.75	0.003883758	0

250501_at	At5g09640	carboxypeptidase - like protein GC donor splice site at exon 11 and 13	0.15	6.68	0.000854174	0
264741_at	At1g62290	aspartic protease	0.15	6.65	3.82257E-06	0
249474_s_at	At5g39190	germin-like protein (GLP2a) copy2	0.15	6.63	0.000323341	0
262528_at	At1g17260	H+-transporting ATPase AHA10	0.15	6.56	1.08261E-05	0
251838_at	At3g54940	cysteine proteinase non-consensus AG donor site at exon 2	0.15	6.55	6.62534E-05	0
248647_at	At5g49190	sucrose synthase	0.16	6.43	2.99763E-06	0
264187_at	At1g54860	unknown protein	0.16	6.28	2.49324E-05	0
253930_at	At4g26740	embryo-specific protein 1 (ATS1)	0.16	6.19	1.08787E-05	0
259167_at	At3g01570	putative oleosin similar to oleosin GB:AAB58402 [Sesamum indicum]	0.17	6.05	4.72677E-06	0
250620_at	At5g07190	embryo-specific protein 3 (ATS3)	0.17	6.03	0.000131087	0
256073_at	At1g18100	terminal Flower 1 (TFL1), putative similar to terminal Flower 1 (TFL1)	0.17	5.95	2.10197E-05	0
249353_at	At5g40420	oleosin ; supported by cDNA: gi_725259_gb_L40954.1_ATHOLEOSIN	0.17	5.90	0.000107705	0
260088_at	At1g73190	tonoplast intrinsic protein, alpha (alpha-TIP)	0.17	5.78	2.35306E-05	0
264482_at	At1g77210	sugar carrier protein, putative similar to sugar carrier protein GI:169735 from [Ricinus communis]	0.17	5.77	0.000271697	0
260716_at	At1g48130	peroxiredoxin identical to SP:O04005 from [Arabidopsis thaliana]	0.18	5.70	0.000281267	0
248297_at	At5g53100	protochlorophyllide reductase; oxidoreductase required for shoot apex development	0.18	5.69	3.84634E-05	0
265672_at	At2g31980	putative cysteine proteinase inhibitor B (cystatin B)	0.18	5.67	0.000846778	0
249112_at	At5g43780	ATP sulfurylase precursor (gb|AAD26634.1)	0.18	5.65	6.44591E-06	0
258240_at	At3g27660	oleosin isoform identical to oleosin isoform GB:S71286 from [Arabidopsis thaliana]	0.18	5.64	3.21882E-05	0
258327_at	At3g22640	unknown protein contains similarity to major storage protein GB:384341 from [Theobroma cacao]; supported by cDNA: gi_16604373_gb_AY058085.1_	0.18	5.61	7.58589E-05	0
254095_at	At4g25140	oleosin, 18.5K	0.18	5.59	7.16342E-06	0
250475_at	At5g10180	sulfate transporter	0.18	5.55	3.29419E-05	0
250157_at	At5g15180	prx10 peroxidase - like protein prx10 peroxidase, Spinacia oleracea, EMBL:SOY16776	0.19	5.34	0.000182366	0
259813_at	At1g49860	glutathione S-transferase	0.19	5.24	0.000219886	0
255741_at	At1g25410	tRNA isopentenyl transferase	0.19	5.19	0.000391423	0

260546_at	At2g43520	putative trypsin inhibitor	0.20	5.06	9.98677E-05	0
263138_at	At1g65090	hypothetical protein predicted by genemark.hmm	0.20	4.97	5.27717E-05	0
252119_at	At3g51030	thioredoxin h	0.20	4.95	0.001359942	0
258578_at	At3g04200	germin-like protein contains Pfam profile: PF01072 germin family	0.20	4.88	2.23269E-05	0
248125_at	At5g54740	2S storage protein-like	0.21	4.79	2.85813E-06	0
253894_at	At4g27150	NWMU2 - 2S albumin 2 precursor	0.21	4.73	6.56441E-07	0
248520_at	At5g50600	11-beta-hydroxysteroid dehydrogenase-like	0.21	4.72	0.000100714	0
265117_at	At1g62500	putative proline-rich cell wall protein (pir|IS52985	0.21	4.72	0.00014669	0
253895_at	At4g27160	NWMU3 - 2S albumin 3 precursor	0.22	4.47	0.00031598	0
259975_at	At1g76470	putative cinnamoyl-CoA reductase similar to cinnamoyl-CoA reductase GB:CAA56103 [Eucalyptus gunnii]; contains non-consensus GG acceptor splice site at exon 4	0.22	4.46	4.15383E-05	0
254036_at	At4g25980	putative peroxidase peroxidase (EC 1.11.1.7) precursor, cationic (clonePNC2) - peanut, PIR2:B38265	0.22	4.46	1.32714E-05	0
265891_at	At2g15010	putative thionin	0.23	4.42	5.11946E-05	0
261181_at	At1g34580	monosaccharide transporter, putative similar to GB:X66856 from [Nicotiana tabacum]	0.23	4.38	0.001620288	2.9392E-186
265095_at	At1g03880	putative cruciferin 12S seed storage protein identical to 12S seed storage protein, gi|808937	0.23	4.35	0.002152607	0
251188_at	At3g62730	putative protein DESSICATION-RELATED PROTEIN PCC13-62 PRECURSOR - Craterostigma plantagineum, PIR:E45509	0.23	4.34	0.000857121	0
260797_at	At1g78390	neoxanthin cleavage enzyme, putative similar to neoxanthin cleavage enzyme GI:9857290 from [Vigna unguiculata]	0.23	4.30	6.21208E-05	0
267431_at	At2g34870	unknown protein ;supported by full-length cDNA: Ceres:29605.	0.23	4.26	1.20527E-05	0
251504_at	At3g59030	putative protein putative multidrug efflux protein NorM - Vibrio parahaemolyticus, EMBL:AB010463;	0.23	4.26	0.000177337	0
266143_at	At2g38905	Expressed protein ; supported by cDNA: gi_15529213_gb_AY052231.1_	0.24	4.24	0.002009474	8.3025E-300
265644_at	At2g27380	hypothetical protein predicted by genefinder and genscan	0.24	4.19	0.000278521	0
248435_at	At5g51210	oleosin-like	0.24	4.15	7.76979E-06	0
264612_at	At1g04560	unknown protein similar to GB:AAC37469; supported by cDNA: gi_15293292_gb_AY051080.1_	0.24	4.12	0.000180422	0
253004_at	At4g38330	putative protein	0.24	4.11	0.013683281	0

248028_at	At5g55620	putative protein similar to unknown protein (gb|AAF04428.1);supported by full-length cDNA: Ceres:27668.	0.24	4.11	0.000202724	0
245072_s_at	At2g23220	putative cytochrome P450	0.24	4.08	6.50859E-05	0
254906_at	At4g11180	putative disease resistance response protein disease resistance response protein 206-d - Pisum sativum, PID:g508844	0.25	3.97	9.47712E-05	0
267317_at	At2g34700	putative proline-rich glycoprotein	0.25	3.96	0.000207292	0
264606_at	At1g04660	unknown protein ;supported by full-length cDNA: Ceres:98340.	0.26	3.92	0.001672704	1.4868E-229
252234_at	At3g49780	putative protein ; supported by full-length cDNA: Ceres: 40302.	0.26	3.88	2.35562E-07	0
266415_at	At2g38530	putative nonspecific lipid-transfer protein ;supported by full-length cDNA: Ceres:18328.	0.26	3.86	5.01269E-05	0
267137_at	At2g23410	hypothetical protein predicted by genscan; supported by cDNA: gi_6651293_gb_AF162441.1_AF162441	0.26	3.83	0.000454657	0
249082_at	At5g44120	legumin-like protein	0.26	3.79	2.31779E-05	0
250583_at	At5g07500	zinc finger transcription factor ; supported by cDNA: gi_2961541_gb_AF050463.1_AF050463	0.27	3.76	0.000935827	0
249495_at	At5g39100	germin - like protein GLP6 exon 2 interrupted by a stop codon, creating non-consensus donor and acceptor splice sites	0.27	3.72	4.88243E-05	0
265725_at	At2g32030	putative alanine acetyl transferase	0.27	3.70	9.36245E-05	0
260928_at	At1g02720	hypothetical protein similar to putative glycosyl transferase GB:CAB80706 GI:7268597 from [Arabidopsis thaliana]	0.27	3.68	4.71511E-05	0
248754_at	At5g47670	putative protein contains similarity to CCAAT-box-binding trancription factor	0.27	3.68	0.000926847	0
267075_at	At2g41070	putative bZIP transcription factor contains a bZIP transcription factor basic domain signature (PDOC00036);supported by full-length cDNA: Ceres:114632.	0.27	3.68	0.000187276	0
266465_at	At2g47750	putative auxin-responsive protein ; supported by cDNA: gi_15810039_gb_AY054288.1_	0.28	3.61	4.07135E-06	0
253767_at	At4g28520	12S cruciferin seed storage protein	0.28	3.60	0.000558273	0
253241_at	At4g34520	fatty acid elongase 1	0.28	3.57	4.35255E-05	0
256464_at	At1g32560	late-embryogenesis abundant protein, putative similar to GI:4102692 from [Glycine max]	0.28	3.55	0.002066597	0
245335_at	At4g16160	pore protein homolog ;supported by full-length cDNA: Ceres:5714.	0.28	3.53	2.88563E-05	0
259742_at	At1g71120	putative proline-rich APG protein similar to ANTER-SPECIFIC PROLINE-RICH PROTEIN APG	0.29	3.42	7.18046E-05	0

		PRECURSOR GB:P40602				
266613_at	At2g14900	similar to gibberellin-regulated proteins	0.29	3.41	3.32565E-05	0
255905_at	At1g17810	tonoplast intrinsic protein, putative similar to tonoplast intrinsic protein GI:21054 from [Phaseolus vulgaris];	0.29	3.41	0.000121925	0
246498_at	At5g16230	stearoyl-acyl carrier protein desaturase stearoyl-acyl carrier protein desaturase sad1 - Linum usitatissimum, EMBL:AJ006957	0.29	3.40	0.000113164	0
267041_at	At2g34315	unknown protein	0.30	3.39	1.25545E-05	0
253904_at	At4g27140	NWMU1 - 2S albumin 1 precursor ; supported by cDNA: gi_13899092_gb_AF370541.1_AF370541	0.30	3.37	0.000183637	0
262644_at	At1g62710	beta-VPE nearly identical to beta-VPE GB:BAA09615 GI:1805364 [Arabidopsis thaliana];	0.30	3.34	0.000518624	0
255302_at	At4g04830	putative protein similar to transcriptional regulator;supported by full-length cDNA: Ceres:9218.	0.30	3.32	0.000766429	0
259142_at	At3g10200	unknown protein similar to ankyrin like protein GB:CAB10215 [Arabidopsis thaliana]	0.30	3.31	0.00029293	0
250519_at	At5g08460	GDSL-motif lipase/acylhydrolase-like protein	0.30	3.29	3.67879E-05	0
265094_at	At1g03890	putative cruciferin 12S seed storage protein highly similar to Brassica napus cruciferin storage protein,	0.31	3.28	5.75905E-05	0
260917_at	At1g02700	unknown protein similar to hypothetical protein GB:CAB80707 GI:7268598 from [Arabidopsis thaliana]; supported by cDNA: gi_15810578_gb_AY056328.1_	0.31	3.26	2.80395E-05	0
251137_at	At5g01300	putative protein several hypothetical proteins - different bacteria	0.31	3.25	6.70745E-07	0
249913_at	At5g22810	GDSL-motif lipase/hydrolase-like protein	0.31	3.24	1.60221E-06	0
262750_at	At1g28710	unknown protein ; supported by cDNA: gi_15027990_gb_AY045852.1_	0.31	3.24	2.03384E-05	0
254494_at	At4g20050	putative protein C65DMY30S	0.31	3.20	0.000225051	0
249867_at	At5g23020	2-isopropylmalate synthase-like protein	0.32	3.17	1.36769E-05	0
248696_at	At5g48360	putative protein similar to unknown protein (gb|AAF02158.1)	0.32	3.17	0.00070063	0
248110_at	At5g55320	wax synthase-like protein	0.32	3.16	0.0005109	0
259782_at	At1g29680	unknown protein	0.32	3.16	0.0127021	1.25537E-80
252123_at	At3g51240	flavanone 3-hydroxylase (FH3) ;supported by full-length cDNA: Ceres:36653.	0.32	3.15	8.74313E-05	0
261305_at	At1g48470	glutamine synthetase, putative similar to PIR:S18603 from [Arabidopsis thaliana];supported by full-length cDNA: Ceres:156550.	0.32	3.14	0.006881798	0

258258_at	At3g26790	transcriptional regulator (FUSCA3) identical to FUSCA3 GB:AAC35247 [Arabidopsis thaliana] (Plant J. 6, 379-387 (1994));	0.32	3.12	0.000151861	0
252605_s_at	At3g45070	sulfotransferase-like protein FLAVONOL 4 -SULFOTRANSFERASE - Flaveria chloraefolia, EMBL:M84136	0.32	3.11	0.009160931	1.1495E-107
264469_at	At1g67100	hypothetical protein similar to hypothetical protein GI:6553940 from [Arabidopsis thaliana]	0.32	3.10	0.000400703	0
246273_at	At4g36700	globulin-like protein	0.32	3.10	0.00628815	3.0815E-137
247762_at	At5g59170	cell wall protein	0.32	3.08	0.000186663	0
266849_at	At2g25940	putative vacuolar processing enzyme identical to GB:D61393; supported by cDNA: gi_16604313_gb_AY058055.1_	0.32	3.08	0.000165402	0
264580_at	At1g05340	unknown protein EST gb|ATTS0295 comes from this gene;supported by full-length cDNA: Ceres:20380.	0.33	3.08	0.000386779	0
261260_at	At1g26680	hypothetical protein predicted by genemark.hmm	0.33	3.03	0.001087115	0
252269_at	At3g49580	putative protein ;supported by full-length cDNA: Ceres:26235.	0.33	3.02	0.006582597	2.71005E-80
252050_at	At3g52550	hypothetical protein	0.33	3.01	9.81134E-05	0
250438_at	At5g10580	putative protein predicted protein, Arabidopsis thaliana; supported by cDNA: gi_14326488_gb_AF385697.1_AF385697	0.33	3.00	0.000890575	0
248918_at	At5g45890	senescence-specific cysteine protease SAG12 identical to senescence-specific protein SAG12 GI:1046373 from [Arabidopsis thaliana]	0.33	3.00	0.000251484	0
251646_at	At3g57780	putative protein	0.34	2.98	0.000805454	0
250258_at	At5g13790	floral homeotic protein AGL15 (sp|Q38847)	0.34	2.98	0.005338602	1.3929E-176
264721_at	At1g23000	hypothetical protein predicted by genscan+	0.34	2.97	0.000114503	0
256898_at	At3g24650	abscisic acid-insensitive protein 3 identical to abscisic acid-insensitive protein 3 GB:Q01593 from [Arabidopsis thaliana], (Plant Cell 4 (10), 1251-1261 (1992))	0.34	2.96	0.00144894	0
261455_at	At1g21070	unknown protein ;supported by full-length cDNA: Ceres:108746.	0.34	2.95	0.000107875	0
264346_at	At1g12010	putative amino-cyclopropane-carboxylic acid oxidase (ACC oxidase) Strong similarity to amino-cyclopropane-carboxylic acid oxidase gb|L27664 from Brassica napus. ESTs gb|Z48548 and gb|Z48549 come from this gene; supported by cDNA: gi_15450652_gb_AY052694.1_	0.34	2.94	4.2419E-06	0

261503_at	At1g71691	hypothetical protein predicted by genemark.hmm, contains Pfam profile: PF00657 Lipase/Acylhydrolase with GDSL-like motif	0.34	2.92	3.73652E-06	0
256827_at	At3g18570	hypothetical protein contains similarity to Pfam profile: PF01277 Oleosin	0.34	2.90	0.000184674	0
245410_at	At4g17220	putative protein	0.35	2.88	0.000308676	0
266578_at	At2g23910	putative cinnamoyl CoA reductase ;supported by full-length cDNA: Ceres:14133.	0.35	2.82	5.13754E-05	0
263753_at	At2g21490	putative dehydrin ;supported by full-length cDNA: Ceres:5256.	0.35	2.82	0.002057671	0
256918_s_at	At3g18960	hypothetical protein similar to hypothetical protein GB:AAC72857 from [Arabidopsis thaliana]	0.36	2.79	0.000417961	0
249702_at	At5g35570	axi 1 (auxin-independent growth promoter)-like protein ; supported by cDNA: gi_14517443_gb_AY039557.1_	0.36	2.78	5.41558E-05	0
248625_at	At5g48880	3-keto-acyl-CoA thiolase 2 (gb|AAC17877.1) ; supported by cDNA: gi_3192892_gb_AF062590.1_AF062590	0.36	2.75	7.23243E-05	0
265108_s_at	At1g62620	similar to flavin-binding monooxygenase (Z71258); similar to ESTs gb|R30018, gb|T23015, and gb|T88100 contains similarity to flavin-containing monooxygenase 2 GB:AAD56413 GI:5923916 from [Mus musculus]	0.37	2.73	0.013530013	1.3552E-132
255471_at	At4g03050	putative oxidoreductase similar to A. thaliana putative protein F21P8.230, GenBank accession number 3445238	0.37	2.71	1.95273E-05	0
257242_at	At3g24220	9-cis-epoxycarotenoid dioxygenase, putative similar to GB:CAB10168 from [Lycopersicon esculentum] (J. Exp. Bot. 47, 2111-2112 (1997))	0.37	2.71	0.000128097	0
247897_at	At5g57810	unknown protein ;supported by full-length cDNA: Ceres:24169.	0.37	2.69	9.73068E-05	0
253827_at	At4g28085	Expressed protein ; supported by cDNA: gi_15028040_gb_AY045877.1_	0.37	2.68	0.000253829	0
262047_at	At1g80160	hypothetical protein predicted by genemark.hmm;supported by full-length cDNA: Ceres:21663.	0.38	2.67	0.000418805	0
258204_at	At3g13960	hypothetical protein predicted by genemark.hmm	0.38	2.64	0.014467774	1.23204E-80
245688_at	At1g28290	prolin-rich protein, putative similar to prolin-rich protein GI:21045 from [Phaseolus vulgaris]	0.38	2.61	0.003251255	3.5237E-49
260961_at	At1g05960	unknown protein similar to hypothetical protein GB:AAF80120 GI:8810459 from [Arabidopsis thaliana]; supported by cDNA: gi_14194168_gb_AF367291.1_AF367291	0.39	2.56	0.000613929	0
266344_at	At2g01580	hypothetical protein predicted by genscan and genefinder	0.39	2.55	0.001791233	0

251540_at	At3g58740	citrate synthase -like protein citrate synthase, cucurbit, PIR:S53007	0.39	2.55	0.000514098	0
253375_at	At4g33280	hypothetical protein	0.39	2.54	0.002683679	3.2281E-228
262740_at	At1g28590	lipase, putative contains Pfam profile: PF00657 Lipase/Acylhydrolase with GDSL-like motif	0.40	2.53	9.91004E-05	0
247421_at	At5g62800	SIAH1 - like protein SIAH1, Brassica napus, EMBL:BNA245479	0.40	2.52	8.82853E-05	0
247904_at	At5g57390	putative protein contains similarity to unknown protein (gb|AAD30633.1)	0.40	2.51	0.004420171	1.6068E-129
261845_at	At1g15960	metal ion transporter, putative similar to metal ion transporter GI:5853313 from [Arabidopsis thaliana]	0.40	2.50	0.00025307	0
256359_at	At1g66460	protein kinase, putative contains Pfam profile: PF00069: Eukaryotic protein kinase domain	0.40	2.49	0.000399601	0
246516_at	At5g15740	putative protein auxin-independent growth promoter - Nicotiana tabacum, PIR:A44226	0.40	2.49	4.79937E-05	0
247182_at	At5g65410	putative protein contains similarity to unknown protein (pir||T05568)	0.40	2.48	0.000151913	0
248365_at	At5g52500	putative protein strong similarity to unknown protein (emb|CAB68146.1)	0.41	2.47	6.51104E-05	0
253575_at	At4g31060	putative protein TINY, Arabidopsis thaliana, PID:E218696	0.41	2.47	0.000222478	0
245576_at	At4g14770	hypothetical protein	0.41	2.46	0.000402784	0
267488_at	At2g19110	putative cadmium-transporting ATPase T20K24.13 has been merged with T20K24.12 per suggestion of Dr. Kristian Axelsen (axe@biobase.dk)	0.41	2.44	0.000209957	0
263982_at	At2g42860	hypothetical protein predicted by genefinder;supported by full-length cDNA: Ceres:42863.	0.41	2.43	0.002423676	7.7801E-177
264494_at	At1g27470	hypothetical protein predicted by genefinder	0.41	2.43	1.0398E-06	0
254506_at	At4g20140	leucine rich repeat-like protein Cf-2.2, Lycopersicon pimpinellifolium, PIR:T10515	0.41	2.42	0.000387724	0
255751_at	At1g31950	terpene synthase family protein, putative contains Pfam profile: PF01397: Terpene synthase family	0.42	2.41	0.005784567	2.3379E-44
263979_at	At2g42840	En/Spm-like transposon protein related to En/Spm transposon family of maize;supported by full-length cDNA: Ceres:41214.	0.42	2.40	0.001817486	0
260091_at	At1g73290	putative serine carboxypeptidase similar to serine carboxypeptidase I precursor GB:P07519 [Hordeum vulgare]	0.42	2.40	0.000824163	0
261729_s_at	At1g47840	hexokinase, putative similar to hexokinase 2 GB:AAB49911 GI:1899025 from [Arabidopsis thaliana]	0.42	2.39	0.002099743	9.94E-273
246241_at	At4g37050	patatin-like protein	0.42	2.39	0.000401979	0
258108_at	At3g23570	unknown protein contains Pfam profile: PF01738 dienelactone hydrolase family;	0.42	2.38	0.001185423	0
265454_at	At2g46530	putative ARF1 family auxin responsive transcription factor	0.42	2.38	4.81802E-05	0

260957_at	At1g06080	delta 9 desaturase identical to delta 9 desaturase GB:BAA25180 GI:2970034 from [Arabidopsis thaliana]	0.42	2.37	0.000796392	0
266824_at	At2g22800	homeodomain transcription factor (HAT9) identical to GB:U09341; supported by cDNA: gi_527638_gb_U09341.1_ATU09341	0.42	2.37	0.000325535	0
255007_at	At4g10020	putative oxidoreductase 11beta-hydroxysteroid dehydrogenase (EC 1.1.1.146) 1 - mouse, PIR1:I56604	0.42	2.35	4.11486E-05	0
256452_at	At1g75240	hypothetical protein predicted by genemark.hmm	0.43	2.35	0.008185277	5.3907E-179
258200_at	At3g13900	putative ATPase similar to ATPase II GB:AAD34706 from [Homo sapiens]	0.43	2.35	0.00338429	7.7959E-168
254447_at	At4g20860	berberine bridge enzyme - like protein ; supported by cDNA: gi_14194126_gb_AF367269.1_AF367269	0.43	2.35	0.000618687	0
265246_at	At2g43050	putative pectinesterase	0.43	2.34	9.92981E-06	0
249866_at	At5g23010	2-isopropylmalate synthase-like; homocitrate synthase-like ; supported by cDNA: gi_12330688_gb_AF327648.1_AF327648	0.43	2.34	0.0015035	0
266391_at	At2g41290	putative strictosidine synthase identical to GB:AF076979; involved in alkaloid biosynthesis	0.43	2.34	2.81029E-05	0
264557_at	At1g09550	putative pectinacetylesterase precursor similar to Vigna radiata pectinacetylesterase precursor, gi|1431629	0.43	2.34	0.000223893	0
248096_at	At5g55240	embryo-specific protein 1; Ca2+-binding EF-hand protein-like	0.43	2.33	0.00279472	3.83397E-29
267053_s_at	At2g38390	peroxidase	0.43	2.32	0.003143035	3.7516E-106
264973_at	At1g27040	nitrate transporter, putative nitrate transporter NTL1 GB:AAC28086 GI:3377517 from [Arabidopsis thaliana]	0.43	2.32	0.000503995	0
247324_at	At5g64190	putative protein strong similarity to unknown protein (gb AAD25673.1)	0.43	2.31	0.00073022	0
257102_at	At3g25050	endoxyloglucan transferase, putative similar to GB:C49539 from [Arabidopsis thaliana] (J. Biol. Chem. 268 (34), 25364-25368 (1993))	0.43	2.31	0.001863255	0
253513_at	At4g31760	peroxidase - like protein peroxidase, Spinacia oleracea, PID:g1781338	0.43	2.31	0.000692576	0
245889_at	At5g09480	PEE-rich protein ;supported by full-length cDNA: Ceres:20407.	0.43	2.31	0.000276025	0
255575_at	At4g01430	predicted protein of unknown function similar to F3D13.3 similar to M. truncatula MtN21, GenBank accession number Y15293	0.43	2.31	0.000465794	0
266654_at	At2g25890	putative oleosin protein	0.43	2.31	0.002449267	2.29299E-50
246550_at	At5g14920	putative protein predicted protein, Arabidopsis thaliana;supported by full-length cDNA: Ceres:32599.	0.43	2.30	0.000419519	0
258498_at	At3g02480	unknown protein similar to pollen coat protein GB:CAA63531 from [Brassica oleracea]	0.44	2.30	0.002314845	5.0684E-204

250794_at	At5g05270	putative protein contains similarity to chalcone-flavonone isomerase (chalcone isomerase)supported by full-length cDNA: Ceres:40439.	0.44	2.29	1.29192E-05	0
263175_at	At1g05510	hypothetical protein similar to unknown protein GI:4105683 from (Oryza sativa)	0.44	2.28	2.13484E-05	0
247610_at	At5g60630	putative protein predicted protein, Arabidopsis thaliana	0.44	2.28	6.58569E-06	0
266770_at	At2g03090	putative expansin Experimental evidence from Dr. Daniel Cosgrove at Penn State Univ. <dCosgrove@psu.edu>	0.44	2.28	0.000252669	0
248545_at	At5g50260	cysteine proteinase similar to cysteine endopeptidase precursor CysEP GI:2944446 from [Ricinus communis]	0.44	2.27	0.000135066	0
256599_at	At3g14760	hypothetical protein predicted by genscan+	0.44	2.27	1.70862E-05	0
262525_at	At1g17060	putative cytochrome P450 41% identical to Cytochrome P450 [Catharanthus roseus] (gi|404690)	0.44	2.27	1.90265E-05	0
252054_at	At3g52540	hypothetical protein ;supported by full-length cDNA: Ceres:99326.	0.44	2.27	0.001032408	0
264891_at	At1g23200	putative pectinesterase similar to GB:AAB57669, location of EST gb|Z35063 and gb|Z35062	0.44	2.26	0.000107861	0
249881_at	At5g23190	cytochrome P450-like protein	0.44	2.25	0.001618102	0
264590_at	At2g17710	unknown protein ;supported by full-length cDNA: Ceres:1011.	0.45	2.24	2.0599E-07	0
255730_at	At1g25460	dihydroflavonol 4-reductase, putative similar to dihydroflavonol 4-reductase GI:1332411 from [Rosa hybrida]	0.45	2.24	0.001046227	0
259812_at	At1g49840	unknown protein	0.45	2.24	0.000684867	0
252312_at	At3g49380	hypothetical protein SF16 protein, Helianthus annus, gb:AAD12670	0.45	2.23	0.000568127	0
266379_at	At2g14690	1,4-beta-xylan endohydrolase	0.45	2.23	0.000660843	0
267337_at	At2g39980	putative anthocyanin 5-aromatic acyltransferase ; supported by cDNA: gi_13937225_gb_AF372968.1_AF372968	0.45	2.23	0.00032598	0
245577_at	At4g14780	kinase like protein	0.45	2.23	7.43883E-06	0
266198_at	At2g38890	unknown protein predicted by genscan and genefinder	0.45	2.21	0.002562738	3.17897E-79
262733_s_at	At1g28670	lipase identical to lipase GB:AAA93262 GI:1145627 [Arabidopsis thaliana] (FEBS Lett. 377 (3), 475-480 (1995)); supported by cDNA: gi_1145626_gb_U38916.1_ATU38916	0.45	2.21	0.00133031	1.5412E-223
263909_at	At2g36490	hypothetical protein predicted by genscan	0.45	2.20	1.26895E-05	0

247953_at	At5g57260	cytochrome P450	0.46	2.19	0.01595322	7.11029E-30
261103_s_at	At1g62900	O-methyltransferase 1, putative similar to GB:AAB96879 from [Arabidopsis thaliana] (Biochim. Biophys. Acta 1353 (3), 199-202 (1997))	0.46	2.19	0.000214119	0
248468_at	At5g50750	UDP-glucose:protein transglucosylase; reversibly glycosylated polypeptide ; supported by cDNA: gi_14326033_gb_AF329280.1_AF329280	0.46	2.18	0.000525874	0
265789_at	At2g01210	putative receptor-like protein kinase	0.46	2.18	0.00011377	0
250611_at	At5g07200	gibberellin 20-oxidase	0.46	2.18	2.92566E-05	0
247780_at	At5g58770	dehydrodolichyl diphosphate - like protein dehydrodolichyl diphosphate, Arabidopsis thaliana, EMBL:ATH277136	0.46	2.17	0.000809376	0
255814_at	At1g19900	unknown protein	0.46	2.17	0.003116764	7.69844E-29
261502_at	At1g14440	hypothetical protein predicted by genemark.hmm	0.46	2.15	0.000713814	0
253099_s_at	At4g37530	peroxidase - like protein peroxidase, Arabidopsis thaliana, gb:X98314	0.47	2.15	0.000294344	0
253346_at	At4g33600	hypothetical protein	0.47	2.14	0.000675726	0
258727_at	At3g11930	ethylene-responsive protein, putative similar to ER6 protein GB:AAD46412 GI:5669654 from (Lycopersicon esculentum)	0.47	2.13	0.000168262	0
256245_at	At3g12580	heat shock protein 70 identical to heat shock protein 70 GB:CAA05547 GI:3962377 [Arabidopsis thaliana]; supported by cDNA: gi_15809831_gb_AY054183.1_	0.47	2.13	0.000329244	0
249289_at	At5g41040	N-hydroxycinnamoyl/benzoyltransferase-like protein ; supported by cDNA: gi_14334525_gb_AY034954.1_	0.47	2.13	0.000944099	0
266322_at	At2g46690	putative auxin-regulated protein	0.47	2.13	0.0058712	1.69402E-20
265615_at	At2g25450	putative dioxygenase ; supported by cDNA: gi_15292706_gb_AY050787.1_	0.47	2.11	0.001419165	1.0314E-227
263734_at	At1g60030	permease, putative similar to permease 1 GI:7844006 from [Zea mays]	0.47	2.11	0.000802591	0
262828_at	At1g14950	major latex protein type1 identical to major latex protein type1 GB:CAA63026 GI:1107493 [Arabidopsis thaliana]	0.48	2.10	0.000174781	0
251293_at	At3g61930	hypothetical protein	0.48	2.10	4.0518E-05	0
255726_at	At1g25530	lysine and histidine specific transporter, putative similar to lysine and histidine specific transporter	0.48	2.10	0.000312751	0

		GI:2576361 from [Arabidopsis thaliana]				
260745_at	At1g78370	2,4-D inducible glutathione S-transferase, putative similar to 2,4-D inducible glutathione S-transferase GI:2920666 from [Glycine max];	0.48	2.09	0.001448447	2.3259E-278
245140_at	At2g45420	unknown protein predicted by genefinder and genscan; similar to gi2347197|AC002338	0.48	2.08	0.000493777	0
256970_at	At3g21090	ABC transporter, putative similar to ATP-binding cassette, sub-family G (WHITE), member 2 GB:NP_036050 from [Mus musculus]	0.48	2.08	0.006503832	1.67821E-15
257869_at	At3g25160	ER lumen protein retaining receptor, putative similar to HDEL receptor required for retention of ER proteins GB:NP_009513 [Saccharomyces cerevisiae]\; contains Pfam profile: PF00810 ER lumen protein retaining receptor	0.48	2.08	9.86036E-05	0
247867_at	At5g57630	SNF1 related protein kinase-like protein ; supported by cDNA: gi_14334389_gb_AY034100.1_	0.48	2.08	0.008999054	1.7796E-104
260299_at	At1g80330	putative gibberellin 3 beta-hydroxylase similar to gibberellin 3 beta-hydroxylase GB:AAC83647 [Arabidopsis thaliana]	0.48	2.08	0.002056645	0
262478_at	At1g11170	hypothetical protein similar to hypothetical protein GB:AAD49998 GI:5734733 from [Arabidopsis thaliana]	0.48	2.08	0.00100865	0
252645_at	At3g44460	bZIP transcription factor -like protein Dc3 promoter-binding factor-1, common sunflower, PIR:T12621;	0.48	2.08	0.000740724	0
248494_at	At5g50770	putative protein contains similarity to oxidoreductase	0.48	2.08	0.001697492	0
249772_at	At5g24130	unknown protein	0.49	2.06	0.00171025	0
253145_at	At4g35560	hypothetical protein	0.49	2.05	0.000422883	0
264387_at	At1g11990	putative growth regulator protein contains similarity to axi 1 gene gb|X80301 from Nicotiana tabacum	0.49	2.04	0.000872369	0
258751_at	At3g05890	low temperature and salt responsive protein (LTI6B) identical to low temperature and salt responsive protein (LTI6B) GB:AAC97511 (Arabidopsis thaliana); supported by cDNA: gi_4325218_gb_AF122006.1_AF122006	0.49	2.03	0.002113697	0
246565_at	At5g15530	biotin carboxyl carrier protein precursor-like protein biotin carboxyl carrier protein (clone BP6) precursor - Brassica napus, EMBL:X90731;supported by full-length cDNA: Ceres:25607.	0.49	2.03	0.00020014	0
247487_at	At5g62150	putative protein predicted protein, Arabidopsis thaliana	0.50	2.02	0.001087691	0
267500_s_at	At2g45510	putative cytochrome P450	0.50	2.02	0.00322842	0

259442_at	At1g02310	(1-4)-beta-mannan endohydrolase precursor, putative similar to (1-4)-beta-mannan endohydrolase precursor GI:9836826 from [Lycopersicon esculentum]	0.50	2.02	0.000215471	0
249729_at	At5g24410	6-phosphogluconolactonase-like protein	0.50	2.01	0.001115931	0
249454_at	At5g39520	expressed protein predicted protein, Synechocystis sp., PIR:S77152;	0.50	2.01	0.001907049	0
267376_at	At2g26330	putative receptor-like protein kinase, ERECTA identical to GB:U47029 and GB:D83257;	0.50	2.01	0.001592232	0
261915_at	At1g65880	amp-binding protein, putative similar to amp-binding protein GI:1903033 from [Brassica napus]	0.50	2.01	0.000488471	0
267131_at	At2g23400	hypothetical protein predicted by genscan	0.50	2.01	0.039250827	1.63084E-90
258873_at	At3g03240	hypothetical protein similar to unknown protein GB:AAD21437 from F2H17	0.50	2.01	0.000976496	0
265511_at	At2g05540	putative glycine-rich protein	0.50	2.00	0.008072751	2.11653E-12

Table S2. The primer sequences used in the present study (the corresponding restriction sites were bold)

	Prime name	The prime sequences （5’-3’）
For qRT-PCR	qACTIN2-F	TGTGCCAATCTACGAGGGTTT
	qACTIN2-R	TTTCCCGCTCTGCTGTTGT
	qUBQ10-F	AGGTACAGCGAGAGAAAGTAGCA
	qUBQ10-R	TAGGCATAGCGGCGAGGCGT
	qCHIL-F	AGTTCACTGCGATCGGAGTT
	qCHIL-R	TCCTCAGCCAAACGATCTCT
	qTT5-F	CATCGATCCTCTTCGCTCTC
	qTT5-R	AGGTGACACACCGTTCTTCC
	qF3H-F	TCAGATCGTTGAGGCTTGTG
	qF3H-R	ATGTCGAAACGGAGCTTGTC
	qF3'H-F	GGCACTAAGCCTCATCGAAC
	qF3'H-R	TTGGCGTCGTGTATTTTCAA
	qDFR-F	AAACGTTAGCGGAGAAAGCA
	qDFR-R	CCTCGTTCCGAGTGATAGGA
	qANS-F	CATCGTGGGTTGGTGAATAA
	qANS-R	GTCCGTGGAGGAAACTTAGC
	qANR-F	GTGACCGGTCTCAAGGAAAT
	qANR-R	ACAGCAAATGTAGCGACCAG
	qFLS-F	ACCGTCATGCGTCAATTACA
	qFLS-R	TCAACGCATCACGCTTTAAC
For protein expression	CHILBF	AAA**GGATCC**ATGGGAACAGAGATGGTCAT (*Bam*H I)
	CHILBF	AAA**GTCGAC**TTAGGTTAAAACTGCGGAGATT (*Sal* I)
	TT5BF	TA**GGATCC**ATGTCTTCATCCAACGCCT (*Bam*H I)

	TT5BR	TT**GTCGAC**TCAGTTCTCTTTGGCTAGT (*Sal* I)
For plant expression	CHILCF	CACCATGGGAACAGAGATGGTCAT
	CHILR	TTAGGTTAAAACTGCGGAGATT
For promoter activity	CHILPF	CACCTGGGGTGGAGTCAAGCATTT
	CHILPR	GAGTGTAAGACTTACTTTCTAAT
For subcellular localization	CHIL-1302-F	TTT**CCATGG**TAATGGGAACAGAGATGGTCAT (*Nco* I)
	CHIL-1302-R	TTT**ACTAGT**GGTTAAAACTGCGGAGATTGA (*Spe* I)
	TT5-1302-F	TTT**CCATGG**TAATGTCTTCATCCAACGCCT (*Nco* I)
	TT5-1302-R	TTT**ACTAGT**GTTCTCTTTGGCTAGTTTTT (*Spe* I)
For yeast two hybrid	yCHIL-F	TTT**CATATG**ATGGGAACAGAGATGGTCAT (*Nde* I)
	yCHIL-R	TTT**CTGCAG**TTAGGTTAAAACTGCGGAGAT (*Pst* I)
	yTT5-F	TTT**CATATG**ATGTCTTCATCCAACGCCT (*Nde* I)
	yTT5-R	TTT**CTCGAG**TCAGTTCTCTTTGGCTAGTT (*Xho* I)
